# Novel method for highly multiplexed gene expression profiling of circulating tumor cells (CTCs) captured from the blood of women with metastatic breast cancer

**DOI:** 10.1186/s12967-023-04242-z

**Published:** 2023-06-26

**Authors:** Morvarid Farhang Ghahremani, Kelly Kai Yin Seto, Woohyun Cho, Michael Craig Miller, Paul Smith, David Frederick Englert

**Affiliations:** 1 ANGLE Biosciences Inc., Toronto, ON Canada; 2 Clinical Development, ANGLE North America, Inc., Plymouth Meeting, Pennsylvania USA

**Keywords:** Parsortix^®^, HyCEAD^™^, Ziplex^™^, CTCs, Breast cancer, MBC, Gene expression

## Abstract

**Background:**

Enumeration of circulating tumor cells (CTCs) has proven clinical significance for monitoring patients with metastatic cancers. Multiplexed gene expression profiling of CTCs is a potential tool for assessing disease status and monitoring treatment response. The Parsortix^®^ technology enables the capture and harvest of CTCs from blood based on cell size and deformability. The HyCEAD^™^ (Hybrid Capture Enrichment Amplification and Detection) assay enables simultaneous amplification of short amplicons for up to 100 mRNA targets, and the Ziplex^™^ instrument quantifies the amplicons for highly sensitive gene expression profiling down to single cell levels. The aim of the study was to functionally assess this system.

**Methods:**

The HyCEAD/Ziplex platform was used to quantify the expression levels for 72 genes using as little as 20 pg of total RNA or a single cultured tumor cell. Assay performance was evaluated using cells or total RNA spiked into Parsortix harvests of healthy donor blood. The assay was also evaluated using total RNA obtained from Parsortix harvests of blood from metastatic breast cancer (MBC) patients or healthy volunteers (HVs).

**Results:**

Using genes with low expression in WBC RNA and/or in unspiked Parsortix harvests from HVs, the assay distinguished between the different breast cancer and ovarian cancer cell lines with as little as 20 pg of total RNA (equivalent to a single cell) in the presence of 1 ng of WBC RNA. Single cultured cells spiked into Parsortix harvests from 10 mL of HV blood were also detected and distinguished from each other. CVs from repeatability experiments were less than 20%. Hierarchical clustering of clinical samples differentiated most MBC patients from HVs.

**Conclusion:**

HyCEAD/Ziplex provided sensitive quantification of expression of 72 genes from 20 pg of total RNA from cultured tumor cell lines or from single cultured tumor cells spiked into lysates from Parsortix harvests of HV blood. The HyCEAD/Ziplex platform enables the quantification of selected genes in the presence of residual nucleated blood cells in Parsortix harvests. The HyCEAD/Ziplex platform is an effective tool for multiplexed molecular characterization of mRNA in small numbers of tumor cells harvested from blood.

**Supplementary Information:**

The online version contains supplementary material available at 10.1186/s12967-023-04242-z.

## Background

Circulating tumor cells (CTCs) found in the blood of cancer patients have demonstrated prognostic value and can provide information regarding tumor type, tissue of origin, stage, and responsiveness to treatment. They provide a relatively noninvasive source of tumor derived material that can be evaluated using a variety of downstream methods, including genomic and transcriptomic analysis using molecular analysis techniques such as RT-qPCR and next generation sequencing, and antigen analysis using immunocytochemistry [1, 2].

There are often very few CTCs in a blood sample, and enriched cell populations typically contain many nucleated blood cells [3]. Various properties of CTCs have been applied to enable their enrichment from blood samples. These include physical properties [4, 5], antibody binding to cell surface markers [6–9], and size, deformability and/or buoyancy in microfluidic and nanotechnology devices [10–14]. The Parsortix^®^ Cell Separation System (ANGLE plc) captures rare cells based on their size and deformability [15].

The presence of CTCs in patients with metastatic breast cancer (MBC) has been correlated with poor prognosis, and the number of CTCs detected in MBC patients has been found to be predictive of disease progression and survival [16, 17]. The presence of CTCs has also been shown to have prognostic value in patients with MBC about to start a new line of treatment [16] and in patients with non-metastatic breast cancer [18]. For patients with metastatic breast cancer, a threshold of five CTCs per 7.5 mL of blood has been used for prognosis [19, 20]. Furthermore, gene expression profiling of CTCs has been used in research and clinical studies in various cancer types such as gastric [21], prostate [22], ovarian [23], pancreatic [24], and breast [25]. In a study by Sieuwerts et al*.* [26]*,* gene expression analysis of cells enriched for CTCs from blood of patients with MBC was found to classify patients with at least five CTCs in a separate tube of blood based on enumeration using immunofluorescent staining, despite the presence of considerable quantities of contaminating leukocytes. Clustering of patients into distinct groups in this study suggested that expression patterns represent different CTC phenotypes with distinct molecular signatures [26].

A meta-analysis of non-metastatic breast cancer patients treated with neoadjuvant chemotherapy indicated that the number of CTCs was a quantitative predictor of outcome, with hazard ratios for survival increasing as the CTC number increased from one to five [19]. Although stochastic variability limits the accuracy of cell enumeration, it is widely accepted that a sensitivity approaching a single CTC is necessary for clinical utility.

Current molecular methods for gene expression analysis are powerful and proven tools, but they can have disadvantages such as inadequate sensitivity, critical dependence on RNA quality, limitations in multiplex capability, high costs and long times to results. Multiplex pre-amplification procedures have been used to overcome inadequate sensitivity and multiplex limitations, but this adds complexity to the workflow and can result in inconsistent, biased amplification [27]. In the current study, we assessed the performance of the HyCEAD^™^ (Hybrid Capture Enrichment Amplification and Detection) method [28] and the Ziplex^®^ instrument [29] for multiplex gene expression profiling of small numbers of target cells in a background of contaminating white blood cells (WBCs). Using breast cancer as a disease model, the performance of an assay measuring the expression levels of 71 genes related to breast cancer and one WBC specific gene (the Landscape^+™^ Breast Cancer assay) was evaluated in: (1) four breast and one ovarian cancer cell lines, (2) Parsortix harvests derived from healthy volunteer (HV) blood spiked with cultured tumor cells or extracted total RNA, and 3) blood samples from patients with MBC. Cell lines with different molecular subtypes (MCF-7: lumina A; BT474-M1: luminal B; SK-BR-3: HER2 positive; MDA-MB-231: triple negative) were used to determine whether small numbers of cells could be distinguished by expression analysis in Parsortix harvests of blood.

Our purpose was to evaluate the utility of the system in highly multiplexed gene expression assays. The method was highly sensitive and provided linear responses for the small tumor cell numbers expected in the blood of many cancer patients. The hands-on time for HyCEAD amplification is less than 1 h (see discussion of workflow in the Methods section) and requires just two enzymes and an aliquot of magnetic beads. The process is designed for the analysis of intact mRNA targets in lysates of living cells such as the CTCs enriched from blood with the Parsortix PC1 system and is well suited for the verification of large numbers of candidate expression markers with potential clinical utility.

## Methods

### Gene-specific primer and ziplex array probe designs

Marker genes with potential relevance to breast cancer, especially in liquid biopsies, were identified from the literature. The genes comprise a model system for testing the performance of the system and assessing the challenges of expression profiling of CTCs enriched from blood. Literature references for the 71 genes are provided in Additional file 1.

Proprietary software was used in a semi-automated process to design sets of primers and probe sets for the 71 breast cancer-related genes, one WBC gene (PTPRC), five synthetic mRNA targets (positive controls for evaluation of the HyCEAD amplification process), six positive (Arabidopsis sequences) and three negative controls (for evaluation of the Ziplex hybridization process) that combined make up the Landscape^+^ Breast Cancer assay. The genes and controls, including the primer and probe sequences, are listed in Additional file 2.

Primer pairs were chosen to amplify approximately 150-base amplicons within regions of specific transcripts with approximately 50% GC content. Primers had similar melting temperatures and lengths between 22 and 38 bases. When possible, exons were selected to amplify all known isoforms of the target genes, and at least one of the primers was chosen to cross an exon junction to avoid amplification of any residual genomic DNA. Target sequences were also chosen to avoid amplification of homologous regions of non-target genes. When possible, the forward gene-specific primers were designed with only two of the possible four nucleotides at the last few bases of their 3’ ends to minimize the potential of primer-dimer formation between different primers. Hybridization probes with uniform melting temperatures were also designed within the amplicons for detection on the Ziplex instrument. Universal primer sequences were appended to the 5’-ends of the primer sequences, and the resulting tailed primers were assessed to avoid oligonucleotides that could form stable hairpin structures.

### HyCEAD (hybrid capture enrichment amplification and detection)

Mixtures of primers were used to amplify the Landscape^+^ Breast Cancer assay target gene and control sequences with the HyCEAD method. Briefly, sample RNA was hybridized with multiple gene-specific reverse primers (RGS primers) containing a universal 5’-tail in a HyCEAD Hybridization mix including 1X Lysis Solution (ANGLE Biosciences Inc.), 1X PBS, and synthetic mRNA controls (ANGLE Biosciences Inc.) at 65 °C for 2 min then at 55 °C for 10 min. Complexes of RGS primers and poly-adenylated mRNA were then captured on Oligo Dynabeads^™^ Oligo(dT)_25_ (ThemoFisher Scientific, Catalog #61005) and incubated at room temperature for 10 min in Bead Binding Buffer (ANGLE Biosciences Inc.). After magnetic separation, excess nucleic acids, other unwanted components in lysates, and unbound RGS primers were washed away using Bead Wash Buffer (ANGLE Bioscience Inc.). After a final washing step, the beads were resuspended in a reverse transcriptase master mix (ANGLE Biosciences Inc.) containing AffinityScript Multiple Temperature Reverse Transcriptase (Agilent, Catalog #600107) and RNasin^®^ Ribonuclease Inhibitor (Promega, Catalog #N2515). Reverse transcription primed by the RGS primers incorporates the universal reverse sequence into the 5’-end of the first strand cDNA, and reverse transcription primed by the oligo-dT on the beads results in strand displacement of the cDNA synthesized with the RGS primer into the medium. The displaced first strand cDNA was recovered in the supernatant and subsequently amplified in a PCR reaction with 5’-phosphorylated universal reverse primers (5′-/Phos/GGAGCACGCTATCCCGTTAGAC-3′) and with gene-specific forward primers (FGS) tailed with a universal priming sequence and a 5’-biotinylated universal forward primer (5′-/Biosg/CGCTGCCAACTACCGCACATC-3′).

**Fig. 1:**
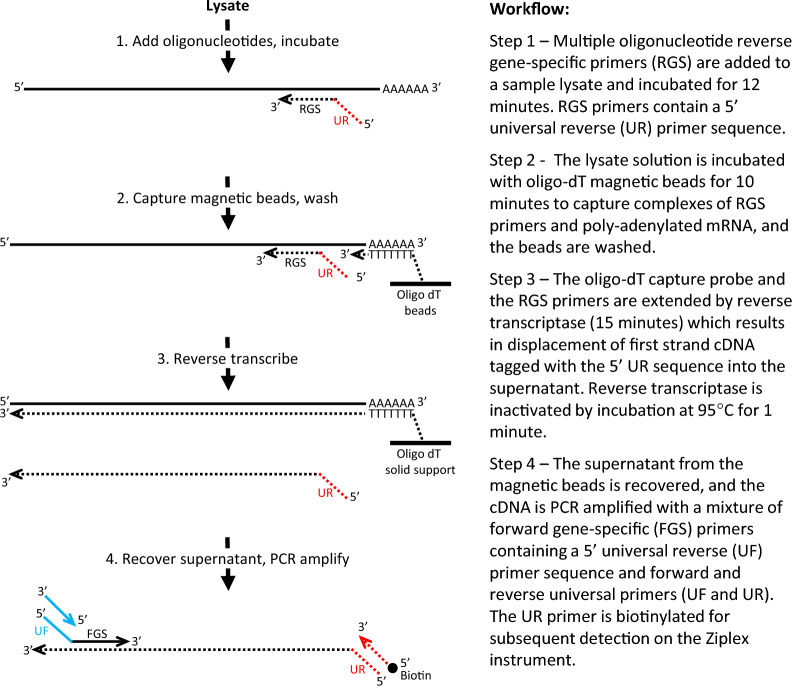
HyCEAD workflow. Integration of the hybridization of reverse transcription primers with purification of poly-A + RNA from crude lysates on magnetic beads removes the need for RNA extraction prior to processing. A large number of reverse primers at high concentrations in the lysate provides favorable hybridization kinetics, and unhybridized primers are washed away along with unbound nucleic acids and other sample components before reverse transcription. This essentially eliminates the interaction of unbound reverse primers during reverse transcription, thereby greatly minimizing the production of primer dimers and off-target artifacts. Although many different gene-specific forward primers are present in solution during PCR amplification, the concentration of the gene-specific primers is relatively low (3.4 nM) to minimize primer-dimer formation, and the universal primers are present at 500-fold greater concentrations. Although primer dimers nevertheless may form between different forward gene-specific primers, they will have the forward universal primer at one end and the complement of the forward universal primer at the other end. As a result, they will form stable hairpin structures which will not be efficiently amplified [30]. These inherent features of the HyCEAD process [28] as well as the design of the gene-specific forward primer (see discussion of primer design above) minimizes the potential interference of primer dimers in the multiplex amplification. The specificity of the process is determined by four levels of selection: capture of poly-A+ RNA, target amplification with two gene-specific primers per target and detection of specific biotinylated amplicons by Ziplex probes that hybridize near the center of the amplicons

For clarity, the workflow of HyCEAD amplification is illustrated in Fig. 1. The time from lysate to PCR setup is 38 min plus pipetting and bead-washing time. The total PCR amplification time is 2.9 h; long annealing times are used for the initial PCR cycles due to the relatively low concentrations of the FGS primers.

### Chemiluminescence-based ziplex gene expression array

Ziplex hybridization was performed using a process adapted from Quinn et al. [28]. Briefly, the phosphorylated anti-sense strands in the PCR reactions (extension products of the universal reverse primer) were digested with Lambda exonuclease (NEB, Catalog # M0262L), and 14 µL of the PCR reaction was combined with 52.5 µL of Hybridization solution B and D (ANGLE Biosciences Inc.) and 6 µL of universal forward primer blocker (5′-GATGTGCGGTAGTTGGCAGCG-3′) at final concentration of 480 nM (ANGLE Biosciences Inc.). The blocker was used to prevent spurious binding of the universal forward primers to the hybridization probes. Target probes were immobilized on nano-porous wafer structures mounted on plastic tubes called TipChips (ANGLE Biosciences Inc.), with each gene specific and control probe being printed in triplicate spots on the TipChip array. The Landscape^+^ Breast Cancer assay TipChips were loaded together with samples and other components of the Landscape^+^ Breast Cancer assay Ziplex kit (ANGLE Biosciences Inc.) in two 96 well plates and placed onto the Ziplex instrument (ANGLE Bioscience Inc.) for automated hybridization and signal assessment. After a 20 min hybridization period and washing, chemiluminescent signals from biotinylated sense-strand PCR products (extension products of the universal forward primer) are acquired with a camera on the Ziplex instrument. Imaging of the chemiluminescent probes arrayed in triplicate on the TipChips and processing of the resulting signal intensity data are performed automatically by the Ziplex instrument. Median values for the evaluable replicate spots of each probe are reported for each sample in tables which can be exported into Excel for analysis or evaluated using various scripts.

### Cell culture

SK-BR-3 and MCF-7 human breast cancer cell lines, the Caov-3 human ovarian cancer cell line, and the A549 human lung cancer cell line were purchased from the American Type Culture Collection (ATCC; Manassas, VA, USA). SK-BR-3, Caov-3, and A549 cells were cultured in McCoy’s 5A medium (Sigma, Catalog #M9309), Dulbecco’s Modified Eagle Medium (DMEM) (Sigma, Catalog #D6429), and F12K medium (Sigma, Catalog #N6658), respectively. All cultures were supplemented with 10% fetal bovine serum (FBS) (Sigma, Catalog #F2442), 100 U/mL of penicillin and 100 µg/mL of streptomycin (Sigma, Catalog #P4333). MCF-7 cells were grown in Eagle’s Minimum Essential Medium (EMEM) (ATCC, Catalog #30–2003) supplemented with 10% FBS, 100 U/mL of penicillin, 100 µg/mL of streptomycin and 10 µg/mL of human insulin solution (Sigma, Catalog # I9278). The cell lines were maintained in a cell culture incubator at 37 °C with 5% CO_2_. For cell culture maintenance, cells were passaged when the confluency reached 70% in T75 flasks. For passaging, the pre-existing culture media in the flasks was aspirated, and the cells were washed 1X with Dulbecco’s Phosphate Buffered Saline (DPBS) without calcium and magnesium (Lonza, Catalog #17–512F). The cells were then dissociated using a 1X Trypsin–EDTA solution (Sigma, Cat #: 59418C) for 3–4 min on a 37 °C hot plate. The trypsin was inactivated with 10 mL of cell line-specific culture media, and the cell solution was collected into a 15 mL Falcon tube. The cells in the solution were enumerated and, depending on the cell line, re-seeded into T75 flasks containing the appropriate media and transferred into a cell culture incubator for continued growth.

### Cell line RNA

Total RNA from different cancer cell lines was either purchased or prepared from cells cultured as described above. Total RNA for the following cell lines was purchased: MDA-MB-231 (ECACC, catalog #92020424), DU145, Skov3, Ovcar8, BT474-M1, PC-3, T-47D, H441 (abmgood, catalog #L542, L537, L540, L591, L544, L523, L517 respectively), and Universal Reference RNAs (UHR) (Stratagene, catalog # 740000). Total RNA was extracted from the A549, MCF-7, SK-BR-3, Caov-3 cell cultures using RNeasy Plus Mini Kit (QIAGEN, catalog #74134). Total WBC RNA was extracted from PBMCs obtained from healthy volunteers using RNeasy Micro Kit (QIAGEN, catalog #74004) following the manufacture’s protocol. Total RNA concentration was measured using NanoDrop ND-1000 Spectrophotometer (Thermo Scientific).

### Parsortix harvests

Peripheral blood collected from healthy volunteers (HVs) into K_2_EDTA Vacutainers was purchased from ZenBio (Durham, NC, USA). The blood samples were processed on Parsortix PR1 Cell Separation Systems (ANGLE plc), previously described by Miller et al*.* [15], within 48 h of collection. Briefly, blood is routed through a disposable microfluidic cell separation cassette with an approximate 6.5 µm-wide critical separation gap (ANGLE plc, Catalog #GEN3P6.5) using controlled and constant pressure conditions (99 mbar). Cells in the blood are captured in the critical gap of the separation cassette based on their size and deformability. By reversing the flow through the separation cassettes, the captured cells were harvested from the cassettes into microcentrifuge tubes in a volume of 210 µL of PBS (Lonza, Catalog #17–512F). An equal volume of 2X lysis solution (ANGLE Bioscience Inc.) was added to each cell harvest and thoroughly mixed. The resulting lysates were spiked with either specific numbers of cultured cells or specific amounts of extracted total RNA and stored at − 80 °C until processing with the Landscape^+^ Breast Cancer assay.

### Harvest spiking

To prepare the contrived samples for analytical verification, a working solution of CellTracker™ Green CMFDA dye (Invitrogen^™^, Catalog #C2925) was prepared by diluting a stock solution in DPBS (Lonza, Catalog #17–512F) at a 1:1000 dilution (final concentration of 6 µM). The media from cultures of SK-BR-3, Caov-3, or MCF-7 cells in in T25 flasks was aspirated, and the cells were briefly washed with DPBS. The DPBS was removed, the working solution of CellTracker Green CMFDA dye was transferred into each T25 flask, and the flasks were gently rocked to ensure complete coverage of the growth surface with the dye solution. The cells were incubated with the CellTracker Green CMFDA dye working solution for 10 min at 37 °C in the CO_2_ incubator. Once the incubation was complete, the dye solution was aspirated, and the flasks were washed with DPBS. The cells were then trypsinized and covered with aluminum foil to prevent photobleaching. Once the cells were detached, complete culture media was added into the flasks to inactivate the Trypsin. The cell suspensions were transferred into 15 mL falcon tubes and centrifuged at 200 × g for 5 min at room temperature. The supernatant was aspirated, and the cell pellets were resuspended in 1 mL of DPBS. The initial cell concentration for each flask was determined, and the cell suspensions were diluted with DPBS to final concentrations of 2 cells/µL. These diluted, pre-labeled cell suspensions were then used for spiking, whereby the approximate number of pre-labeled cells suspended in DPBS were deposited onto the inner wall of empty microcentrifuge tubes. The fluorescently labeled cells spiked into each microfuge tube were enumerated under the FITC channel of an inverted fluorescence microscope (Leica, DMi8) to determine the exact number of cells contained in each tube. The cells captured by the Parsortix^®^ PR1 system from the HV blood samples were harvested directly into the spiked microfuge tubes, and the harvests were used to wash the spiked cells down the side of the tube. An equal volume (i.e., a 1:1 ratio) of 2X lysis solution (ANGLE Biosciences Inc.) was then added to each spiked harvest, and the tubes were vortexed to mix the cells with the lysis solution. The tubes were then spun briefly in a microcentrifuge to collect the lysates at the bottoms of the microfuge tubes. The lysates were stored frozen at − 80 °C until further processing using the Landscape^+^ Breast Cancer assay.

### Patient samples

Whole blood samples from MBC patients (women with either newly diagnosed metastatic breast cancer who were about to start a new line of therapy of any type for the treatment and/or management of their disease or those with currently progressive or recurrent disease as determined by any means) as well as from a control population of healthy female volunteers (HVs, women with self-declared no prior/current history of cancer and no known history of breast disease) were collected under the ANG-002 HOMING study (NCT ID# NCT03427450). All study subjects provided written informed consent prior to participation in the study. All laboratory testing was performed by operators blinded to the clinical status of the subjects. The samples used in this evaluation were processed in the laboratory of Julie Lang, M.D. (Cleveland Clinic, Lerner Research Institute, US) as previously described by Lang et al*.* [31], Kaur et al*.* [32] and Cohen et al. [33]. Briefly, blood collected into 10 mL K_2_EDTA tubes was processed within 8 h after collection on Parsortix^®^ PC1 System (now an FDA cleared device, DEN200062). For each sample, the population of cells captured in the separation cassette were harvested directly into a 0.2 mL PCR tube. The harvest was then centrifuged at 400 × g for 5 min at room temperature, and as much of the supernatant as possible was removed without disturbing the cell pellet. The cell pellet was then resuspended in 10 µL of SideStep Lysis and Stabilization Buffer (Agilent, Catalog #400900). Samples were stored at − 80 °C until further processing. A portion of the lysates generated by the Lang lab were used for RNA-sequencing. The remaining portions of the lysates were then shipped to ANGLE Biosciences Inc. on dry ice, where 2 µL of each lysate was mixed with 2X lysis solution and further processed using the Landscape^+^ Breast Cancer assay as explained above.

### Data analysis and hierarchical clustering

The R language [34] (version 4.1.2) was used for analysis of the gene expression data, including the following packages: Pheatmap, Factoextra, Beeswarm, openxlsx.

## Results

### Primer/probe set screening

For each of the target genes listed in Additional file 2, three primer/probe sets were designed and three pools of primers and probes were tested with 50 pg of total RNA from a battery of up to twelve cell lines (MDA-MB-231, DU145, Skov3, Ovcar8, BT474-M1, PC-3, T-47D, H441, A549, MCF-7, SK-BR-3, Caov-3), Universal Human Reference RNA (UHR), and 1 ng of total RNA from WBCs. Responses to cell line test samples of pairs of primer/probe sets (three plots per gene) are shown in Additional file 3. Primer/probe sets for six of the genes (which had been previously screened for an ovarian cancer assay [35]) were screened with six samples, and 59 primer/probe sets were screened with fifteen samples. Only a single primer/probe set was designed for three candidate genes, so they were not screened in this manner. By visual inspection of these data, one primer/probe set was chosen for each target gene. Some genes were eliminated from further evaluation due to very high expression in WBCs or very low expression in all cell lines. In the majority of cases, the responses of at least two of the three primer/probe sets for a given gene appeared to be correlated with each other (although with substantially different intensities in some cases), indicating that the different primer/probe sets responded to the same transcripts. We chose primer/probe sets that had high signal intensities for the cell line RNA (particularly breast cancer cell lines) compared to the signal intensities observed in the WBC RNA and that appeared to be correlated with at least one other primer/probe set for the gene. The chosen primer/probe sets were combined into a single model assay (71 breast cancer genes and the WBC marker PTPRC) (the Landscape^+^ Breast Cancer assay) for evaluation of the gene expression method.

### Gene expression can distinguish between breast cancer cell line phenotypes

To test the sensitivity of the Landscape^+^ Breast Cancer assay, the gene expression levels using different concentrations (20, 40, 60, 80, and 100 pg) of total RNA from four different breast cancer cell lines (BT474-M1, MDA-MB-231, SK-BR-3 and MCF-7) and one ovarian cancer cell line RNA (Caov3) were measured in triplicate at each RNA concentration. The 20 pg RNA level represents the approximate amount of total RNA contained in a single cell [36].

**Fig. 2:**
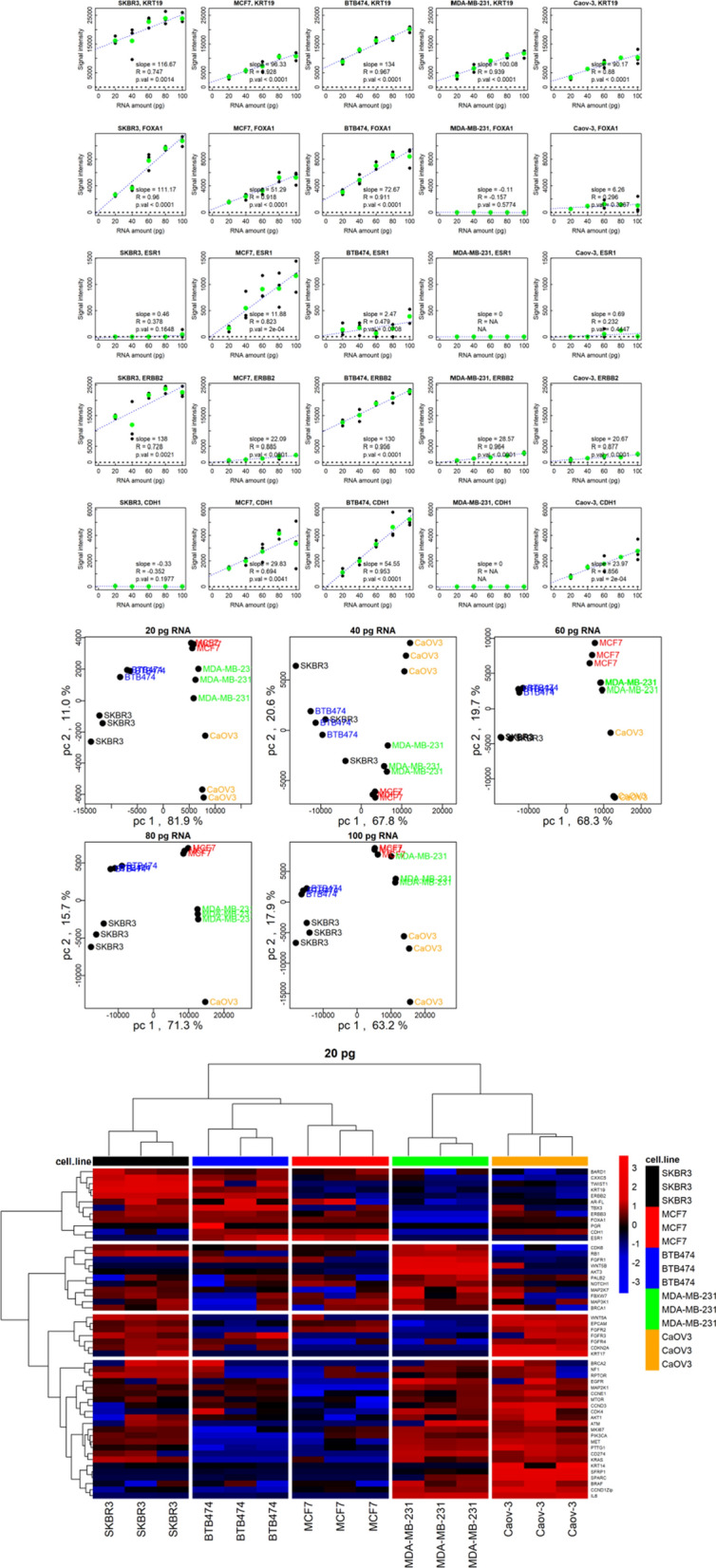
Gene expression analysis of five cancer cell lines with five total RNA concentrations. **A** Responses to increasing amounts of total RNA from five different cancer cell lines for probes to five different genes. In the absence of cell line RNA (no template control samples with 0 pg of total RNA), there were only very small signals on the probes. For each probe the medians of nine no template control samples were subtracted from the cell line data to obtain net signals. The gene symbols and the cell lines are indicated at the top of each plot. The assay was performed in triplicate using separate aliquots of total RNA. Individual replicates are plotted in black, and the mean values of the three replicates are plotted in green. Regression lines were fit to the mean values, and the slopes of the regression lines are indicated on the plots. The correlation coefficients (R) and the p-values of the correlation coefficients are also indicated on the plots. Note that in some cases, 20 pg of RNA produced very large signals (nearing saturation), indicating high expression of the gene within certain cell lines. **B** Principal Components Analysis of the data from 53 genes assayed with five different amounts of total RNA from five different cell lines. The data were mean centered but not scaled, so some genes may have had more influence on clustering than others; clustering of the cell lines was somewhat better without scaling. **C** Hierarchical clustering of the data from 53 genes assayed with 20 pg of total RNA from five different cell lines. Data were log2 transformed, and then the data for each probe in the columns were mean centered and scaled by dividing by the standard deviation for each probe. Colors represent relative Z-Scores. The distance method used for hierarchical clustering was non-parametric rank order Kendall correlation, and the linkage method used was Ward.D2

The expression levels of the probes varied between the different cell lines and were linearly related to the amount of total RNA up to 100 pg, except in some cases in which the response of the Ziplex instrument was saturated (e.g., KRT19 or ERBB2 in the SKBR3 cell line). Examples of the expression levels for five of the probes in the five cell lines are shown in Fig. 2A. Plots of the responses of all the breast cancer genes are shown in Additional file 4. The assay was performed in triplicate (i.e., each multiplex assay was repeated with separate aliquots of total RNA in the lysis solution). For some genes, differences of up to two-fold in the signal intensities were observed between the replicates; this difference would correspond to about one Ct in an RT-qPCR assay.

The slopes, correlation coefficients and p-values of the correlation coefficients of the signal intensity responses to the amount of each cell line RNA in the assay (see annotations in Fig. 2A) were tabulated for each of the primer/probe sets (Additional file 4**)**, excluding two genes, CXCL9 and WNT1, which had essentially no signal in these samples. The limit of detection was also calculated for each gene and each cell line (Additional file 4); the median limit of detection of the probes was 0.41 pg of total RNA (range of 0.006–1300 pg). A slope greater than one indicates that a single cell (~ 20 pg) of the respective cell line would produce a signal intensity of ~ 20 units. The detection limit for most of probes was less than 20 pg (Additional file 4), so single cells were potentially detectable in the absence of background interference. A correlation coefficient greater than 0.65 indicates a likely significant relationship between the observed response and the amount of RNA; all of the p-values of correlation coefficients greater than 0.65 were less than 0.05. A total of 53 (74%) of the 72 probes had slopes greater than one and a correlation coefficient greater than 0.65 for at least one of the five cell lines tested, and were therefore considered to be potentially informative for the detection of a single cell.

Principle Components Analysis (PCA) was performed using the expression levels from the 53 potentially informative probes (Fig. 2B). The principal components distinguished between the cell lines in most cases for all five amounts of RNA, corresponding to a range of approximately one to five cells.

Hierarchical clustering was also performed on the cell line data for each amount of cell line RNA. The heatmap for the results obtained using 20 pg of RNA is shown in Fig. 2C. The heatmaps for all five amounts of RNA and the raw data are shown in Additional file 5. The replicates of each cell line clustered together irrespective of the total RNA concentration and were distant from each other based on the dendrimers at the top of the heatmaps. There were four predominant clusters of correlated genes that were differentially expressed between the cell lines, reflecting the different phenotypes of the cell lines representing different breast cancer subtypes and ovarian cancer: MCF-7 (luminal A), BT474-M1 (luminal B), MDA-MB-231 (TNBC), SK-BR-3 (HER2 +), Caov-3 (ovarian cancer). Thus, phenotypes of cell lines were distinguished by the Landscape^+^ Breast Cancer assay with as little as 20 pg of total RNA (equivalent to a single cell) by groups of correlated genes.

### Breast cancer cell line phenotypes can be distinguished in the presence of WBC background

**Fig. 3:**
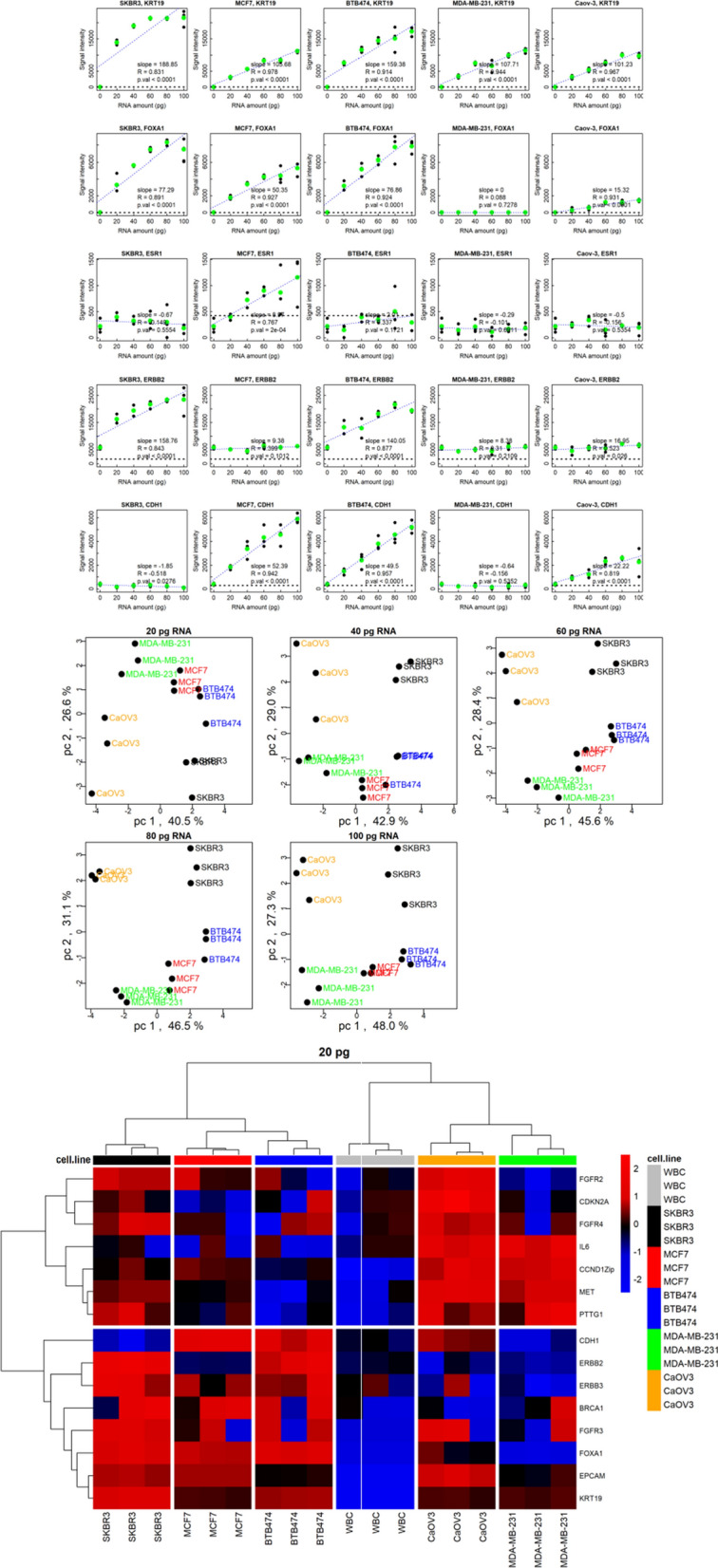
Gene expression analysis of five cancer cell in the presence of WBC background. **A** Responses to increasing amounts of total RNA from five different cell lines for probes to four different genes in the presence of 1 ng of WBC total RNA. For each probe the medians of nine no template control samples were subtracted from the cell line data to obtain net signals, and the results are plotted as in Fig. 2A. **B** Principal Components Analysis of the data from 15 genes assayed with five different amounts of total RNA from five different cell lines in a background of 1 ng of WBC RNA. The data were mean centered and scaled for clustering. **C** Hierarchical clustering of the data from 15 genes assayed with 20 pg of total RNA from five different cell lines in a background of 1 ng of WBC RNA. Data were log2 transformed, and then the data for each probe in the columns were mean centered; the data were not scaled, so some genes may have had more influence on clustering than others. The distance method used for hierarchical clustering was non-parametric rank order Kendall correlation and the linkage method used was Ward.D2

To challenge the specificity of the Landscape^+^ Breast Cancer assay in a controlled experimental setting, we repeated the above experiments with the modification of adding 1 ng of WBC total RNA. As expected, presence of WBC RNA added background signals to many of the probes due to expression of the genes in the blood cells. The expression levels of the same probes as in Fig. 2A are shown in Fig. 3A with the WBC background. Plots of the responses of all the breast cancer genes in the presence of WBC background are shown in Additional file 6. Despite the significant expression of ERBB2 in WBCs, there was a measurable response on the ERBB2 probe for two of the cell lines (SK-BR-3 and BTB474-M1) in the presence of WBC RNA. However, the low-level expression observed in Fig. 2A for the other three cell lines was obscured by the substantially greater signal from the WBCs (Fig. 3A). Expression of ESR1 was also obscured by the WBC background in most of the cell lines but was potentially detectable with a few MCF7 cells. On the other hand, the responses of the probes for CDH1 and FOXA1 were not impacted by the presence of WBC RNA.

The slopes, correlation coefficients and p-values of the correlations of the signal intensity responses to the amount of cell line RNA in the assay (see annotations in Fig. 3A) were tabulated for each of the primer/probe sets (Additional file 6). Compared to the results without WBC background (Additional file 4) there were fewer genes with significant slopes are low detection limits.

The primer/probe sets for genes with slopes and detection limits indicating that they were responsive to small amounts of cell line RNA, and for which a student’s t-test indicated that there was a significant difference (p < 0.05) in the mean signal intensity between replicates of samples with only WBC RNA and replicates of samples with 20 pg of at least one of the cell line RNAs, were selected. The 15 probes that met these criteria were used for principal components analysis (Fig. 3B). The first two principal components of variance distinguished between the cell lines at all RNA levels.

The heatmap for hierarchical clustering on the cell line data with these 15 probes when 20 pg of cell line RNA was used in the assay is shown in Fig. 3C. There were two major gene clusters that separated the luminal and HER2 positive breast cancer cell lines from the triple negative breast cancer line and ovarian cancer cell line. These 15 genes were able to distinguish the five cell lines from un-spiked WBC RNA as well as from each other when as little as 20 pg of cell line RNA was present in a background of 1 ng of WBC RNA. The heatmaps and the raw data for all five amounts are shown in Additional file 7.

### Breast cancer cell line phenotypes can be distinguished in Parsortix harvests of healthy volunteer blood

There will inevitably be variability in the quantity and heterogeneity of WBCs present in Parsortix harvests from cancer patient blood, and this variability will have an impact on the signals from genes expressed in WBCs that may be greater than the signals from CTCs. To assess the effect of this variability and mimic conditions of clinical samples, we evaluated the ability of the Landscape^+^ Breast Cancer assay to detect the presence of cancer related genes in lysates Parsortix harvests obtained from the blood of healthy volunteers (HVs) spiked with either known numbers of cells or total RNA from cultured tumor cell lines. Parsortix harvests were analyzed without spikes or with spikes of either (1) two cells of MCF-7, SK-BR-3, or Caov-3 cell lines obtained directly from cell cultures or (2) 50 pg of total cell RNA of the same cell lines that were added individually. Only half of the volume of the harvests spiked with two cells was used for processing with the Landscape^+^ Breast Cancer assay, so the signal intensity values obtained were representative of approximately one cell. Overall, the signal intensities from the samples spiked with 50 pg total RNA were approximately 2.5 times higher compared to those obtained from the samples spiked with two cells (for which only 50% of the lysate volume was assayed), providing further evidence that the approximate amount of total RNA in a single tumor cell is 20 pg.

**Fig. 4:**
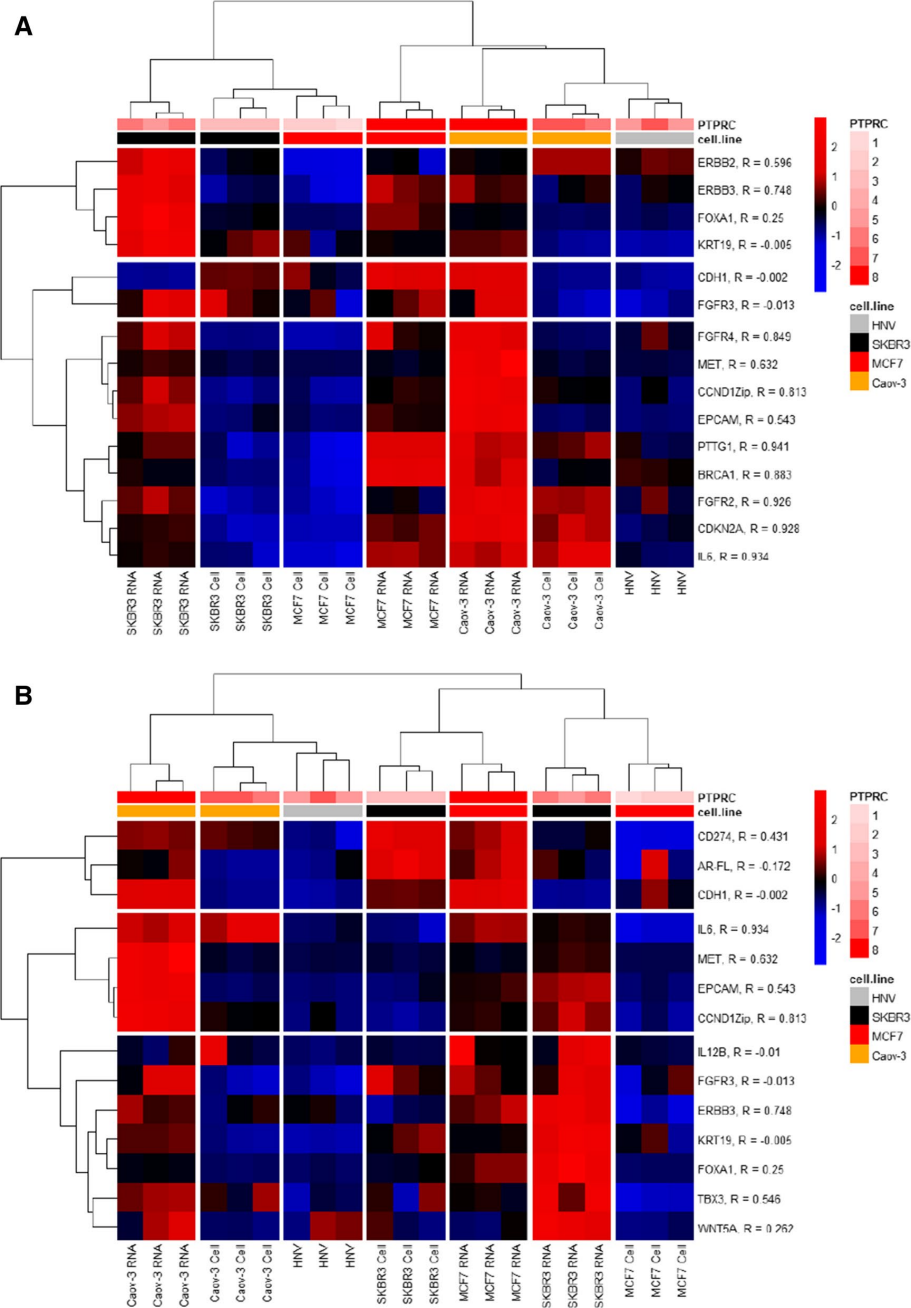
Gene expression analysis of Parsortix harvests from HV blood without or with cancer cell spikes **A** Heatmap of hierarchical clustering for 15 genes in Parsortix harvests from HV blood spiked with either a single cell line cell or 50 pg of cell line RNA. The 15 genes shown are the same as those shown in Fig. 3C. The data were mean centered and scaled, the colors indicate relative Z-scores. The distance method for hierarchical clustering used was non-parametric rank order Kendall correlation and the linkage method used was Ward.D2. The “PTPRC” annotation at the top of the heatmap and the scale of 1 to 8 to the right of the heatmap indicate the level of PTPRC expression in the samples (a range of net Ziplex probe intensities of 565–8200). The correlation coefficient (R) between the expression of individual genes and the WBC marker PTPRC is indicated in the annotations to the right of each gene name. All the nine genes in the bottom gene cluster were well correlated with PTPRC. **B** Heatmap of hierarchical clustering for 14 potentially informative genes in Parsortix harvests from HV blood spiked with either a single cell line cell or 50 pg of cell line RNA. The 14 genes had at least two-fold-differences between unspiked and at least one spiked sample and had relatively small p-values. The data processing and annotation is the same as in A. The raw data used for hierarchical clustering are in Additional file 8

The heatmap of hierarchical clustering for the fifteen genes previously found to distinguish between the five cell lines in a uniform WBC background and un-spiked WBC RNA as well as from each other in the spiked and unspiked HV Parsortix harvests is shown in Fig. 4A. The expression of the gene PTPRC (transcript for the WBC protein CD45) provides an estimate of the number of WBCs present in the samples. PTPRC expression varied considerably between samples, as indicated by the annotation at the top of the heatmap. Genes with expression that correlates well with the expression of PTPRC are likely to indicate the presence of the genes in WBCs rather than or in addition to their presence in tumor cells. The correlation of each of the fifteen genes with PTRC is indicated in the annotations at the right side of the heatmap. Nine of the genes were in a major cluster in which the expression levels for all genes in the cluster corresponded closely to the level of PTPRC in the samples. The expression levels for 4 of the 6 genes in the other two clusters did not correspond closely with PTPRC expression. Expression of the genes in WBCs does not necessarily preclude their potential as CTC markers, although it will depend on their level of expression in the tumor cells. Although the classification of the samples in Fig. 4A corresponded to the presence of the spiked cells, it was also highly associated with the number of WBCs in the harvests (PTPRC bar at top of heatmap in Fig. 4A). The raw data for Fig. 4 are in Additional file 8.

A different set of primers/probes for 14 genes that had at least two-fold differences between un-spiked harvests and harvests spiked with cells or total RNA for at least one cell line and with smaller p-values (p between 0.05 and 0.1) were chosen as potentially informative makers and used for hierarchal clustering **(**Fig. 4B**)**. Although the expression of some of these genes correlated well with PTPRC, the expression patterns generally corresponded with the samples spiked with a specific cell line or cell line RNA. For example, the samples spiked with SK-BR-3 cells and those spiked with MCF-7 cells had relatively low expression of PTPRC, but the expression levels of the genes in the upper three-gene cluster (with poor correlation to PTPRC expression) were very different. The mean values for PTPRC expression of the samples spiked with SK-BR-3 RNA and the un-spiked HV samples differed by less than 3%, but the expression of the genes in the center four-gene cluster was greater in the spiked samples, even though all four of these genes were well-correlated with PTPRC. The samples spiked with Caov-3 RNA and with MCF-7 RNA had high levels of PTPRC expression, but they clustered very far from each other with great differences in expression of the center four-genes cluster despite the high correlation with PTPRC.

These results demonstrate that with appropriate gene selection (high expression in tumor cells and relatively low expression in WBCs), it is possible to detect single cancer cells in Parsortix harvests and distinguish between different cancer types even with substantial variation in WBC contamination in the harvests.

### Expression markers to identify MBC patients with CTCs in Parsortix harvests in the presence varying WBC contamination

Gene expression analysis using RNA-Seq on total RNA isolated from Parsortix harvests of blood samples from patients diagnosed with MBC has been shown to provide clinically relevant information on tumor biology and potential identification of predictive CTC biomarkers [37]. Since the Landscape^+^ Breast Cancer assay was shown to be able to detect and distinguish cancer cells in contrived samples, the assay was evaluated using clinical samples. Small portions (20%) of the Parsortix harvest lysates obtained from 15 MBC patients and 15 HVs enrolled in the ANG-002 HOMING study with similar ages and menopausal status were processed using the Landscape^+^ Breast Cancer assay (Additional file 9**)**.

Quality of the HyCEAD/Ziplex procedure was controlled using synthetic mRNA and Arabidopsis controls and standard acceptance metrics for these controls. All samples evaluated using the Landscape^+^ Breast Cancer assay had acceptable signal intensity levels for all of the HyCEAD (synthetic mRNA) and Ziplex (Arabidopsis) control probes and were therefore included in all of the subsequent analyses.

**Fig. 5:**
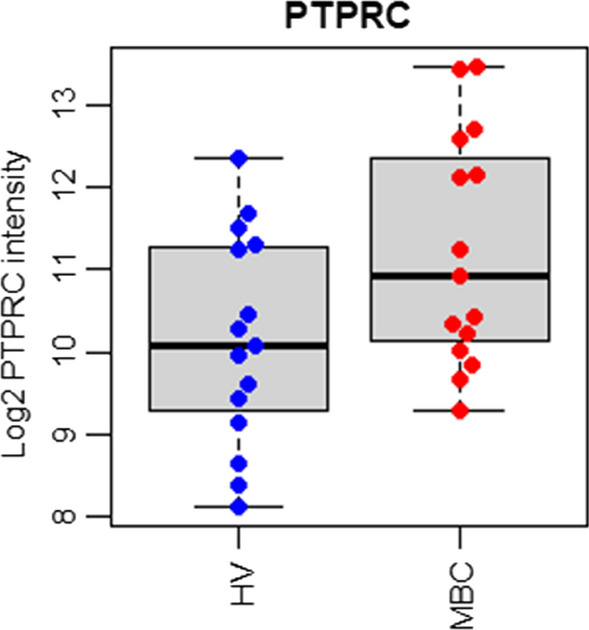
Expression of the PTPRC gene in Parsortix harvests from HVs and MBC patients. Mean signal intensity of the PTPRC probe in the Parsortix harvests from MBC patients was significantly higher compared to the Parsortix harvests from HVs, indicating that the Parsortix harvests from the MBC patients contained higher numbers of WBCs

The expression of the PTPRC gene was significantly higher in the MBC patients compared to the HVs (Fig. 5). The mean signal intensity of the PTPRC probe in the samples from the MBC patients was 2.3 times higher compared to the HV samples (p = 0.05), indicating that the Parsortix harvests from the MBC patients contained more WBCs (and possibly different cell types). The expression of genes present in WBCs may therefore be significantly higher in the MBC patient samples simply due to the increased number of WBCs and independent of the presence of CTCs.

**Fig. 6:**
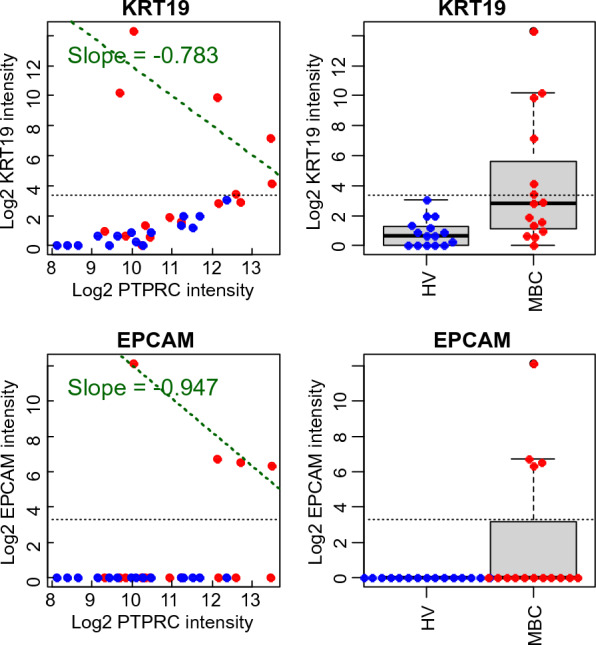
Expression levels of KRT19 and EPCAM in Parsortix harvests from HVs and MBC patients. The log-transformed signal intensities of KRT19 and EPCAM are correlated to the log-transformed signal intensities of PTPRC for the HVs and the MBC patients (left side), and the expression levels are compared between the HV and MBC patients in combination boxplot/beeswarm plots (right side). The HVs are plotted in blue, and the MBC patients are plotted in red. The horizontal dotted line is drawn at an arbitrary cutoff of ten units. The green regression lines were fit only to samples with expression levels of KRT19 or EPCAM greater than the arbitrary threshold of ten units indicated by the horizontal dotted lines. The negative slopes of the PTPRC regression lines merely indicate that there was no meaningful correlation with PTPRC due to the small number of samples above the arbitrary threshold

The genes used in the Landscape^+^ Breast Cancer assay were ranked according to the relationship between the expression level of the specific gene and the expression level of PTPRC. Genes for which the expression levels had positive slopes and relatively good correlation with PTPRC are likely to be expressed in WBCs, and thereby reflect the number of WBCs in the harvests. The genes were sorted with increasing slopes for the samples with intensity levels greater than an arbitrary threshold level of ten expression units. The expression levels of all the breast cancer genes vs. PTPRC are plotted in Additional file 10. The negative slopes observed for genes at the top merely reflect the fact that there were few samples with expression levels above the threshold, and the slopes indicate a lack of a demonstrable correlation with PTPRC. Genes near the bottom, however, are strongly correlated with PTPRC and are not likely to be informative for CTCs. Genes at the top of the list were expressed at a level above the arbitrary threshold exclusively in the MBC patient samples. Two examples are shown in Fig. 6. Some genes (e.g., KRT19 as shown in Fig. 6) appear to be expressed at low levels in WBCs in both the HVs and MBC patients as reflected in the correlation between the expression of the gene and the expression of PTPRC. The correlation coefficient of the data points with KRT19 expression less than or equal to the arbitrary cutoff of ten units was 0.935(*p*-value = 2.3 X 10^–11^); based on the data in Additional file 4 the detection limit for KRT19 is about 0.01 pg of cell line total RNA or a signal intensity of about one unit. However, KRT19 was expressed at much higher level in four MBC patients than in all the other subjects (p-value = 0.0075). There was no detectable expression of EPCAM in the HV samples or in the majority of the MBC patient samples, but the there was substantial expression in four MBC patients. Some genes (e.g., EGFR, Additional file 10) were expressed at substantially higher levels on average in the MBC patient samples compared to the HV samples, but there was no significant correlation with the expression of PTPRC (a slope of about zero).

**Fig. 7:**
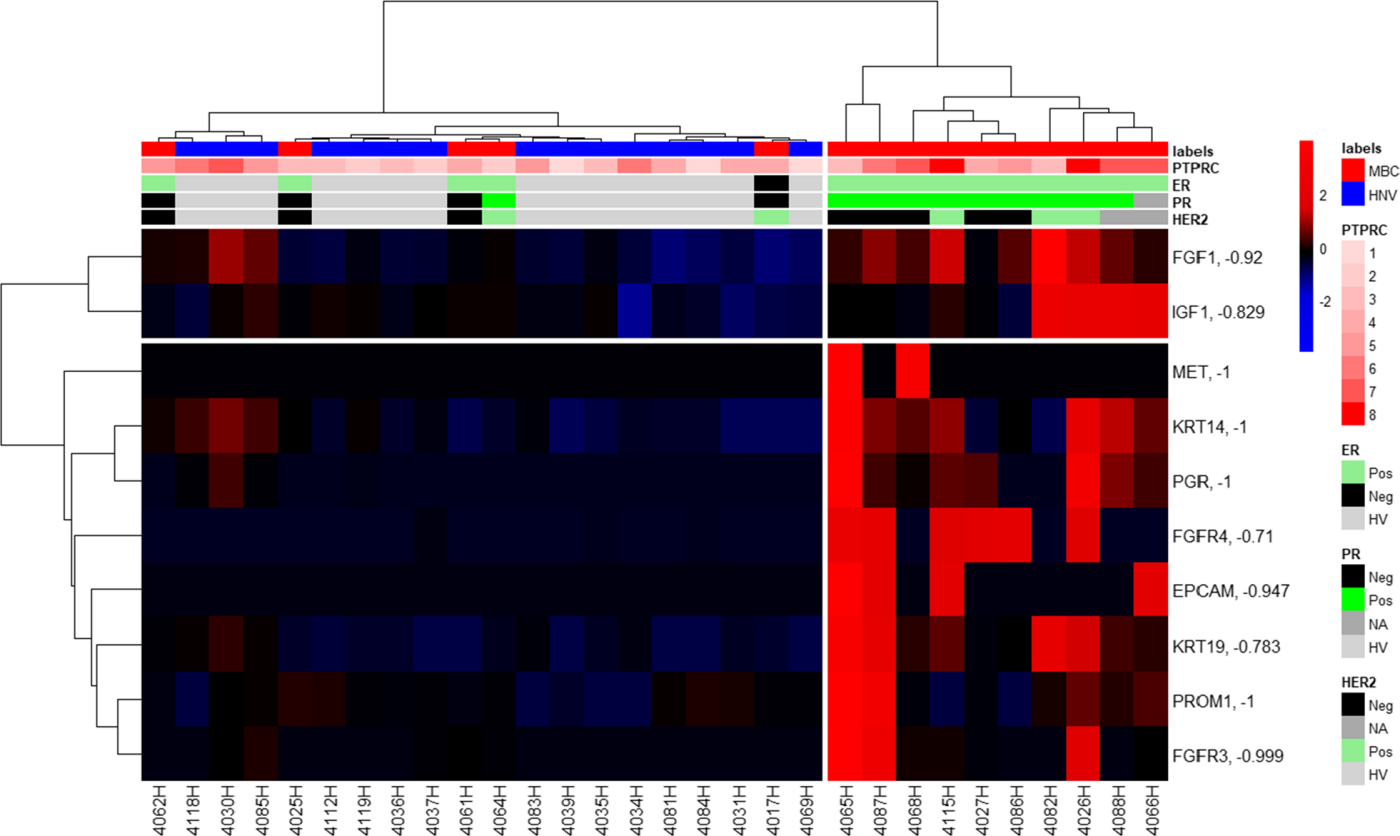
Hierarchical clustering of expression of 10 genes in Parsortix harvests from HVs and MBC patients. The “labels” annotation across the top of the heatmap indicates results from HVs (blue) and MBC patients (red). The “PTPRC” annotation at the top of the heatmap and the scale of 1 to 8 to the right of the heatmap indicate logarithmic scaling of the level of PTPRC expression in the samples, which ranged from 278 to 11,300. The correlation coefficient (R) between the expression of individual genes and the WBC marker PTPRC is indicated in the annotations to the right of each gene name. Additionally, the ER, PR and HER2 assessments of the MBC patient’s tumors using results obtained from a review of their medical records are indicated at the top of the heatmap. Data were log2 transformed, and then the data for each probe in the columns were mean centered and scaled by dividing by the standard deviation for each probe. Colors represent relative Z-Scores. The distance method for hierarchical clustering was Euclidean, and the linkage method was Ward.D. The raw data are provided in Additional file 11.

Hierarchical clustering (Fig. 7) of the ten genes at the top of the list (exclusively expressed at levels above the threshold level of ten in almost all cases in the MBC patients) resulted in two clusters, one of which contained two thirds of the MBC patients and no HVs. Five of the MBC patients clustered with the HVs in this evaluation. The samples from these MBC patients may be those that did not have CTCs in the Parsortix harvests; it has been observed that some MBC patients (35%) do not have detectable CTCs in their peripheral blood [38]. Four of these five MBC patients were PR-negative based on histopathology of their tumors whereas the patients in the major MBC cluster all had PR-positive tumors. The expression levels of the ten genes were very heterogeneous; in many cases only a couple of the MBC samples expressed the genes at levels above the arbitrary threshold (Additional file 10). This reflects the well-known heterogeneity of CTCs [39], but also suggests that a simple algorithm with multiple genes with near-exclusive expression above threshold levels in the heterogeneous population of tumor-derived CTCs might be able to be used to identify samples that contain CTCs in Parsortix harvests. These genes, along with other informative markers, could also potentially be used to assess the phenotypes of the CTCs in the Parsortix harvests.

## Discussion

To realize the potential of CTCs obtained from a liquid biopsy for the evaluation of the molecular status of a cancer patient's disease, very sensitive and specific assays are required. Gene expression analysis can provide genotypic information about a patient’s tumor during the evolution of the disease. Given the molecular heterogeneity of tumor-derived CTCs, multiplexed assays assessing many different genes may be necessary for the accurate characterization of the disease. The results presented here indicate that the Landscape^+^ Breast Cancer assay provides single-cell sensitivity and adequate specificity to distinguish between cell lines representative of the major molecular subtypes of breast cancer. Gene expression analysis, in conjunction with other modes of molecular analysis, may enable assessment of disease progression and guidance for selection of the most effective therapy during the course of a patient’s disease.

The HyCEAD target capture and amplification procedure provides a relatively simple workflow for highly multiplexed gene expression analysis. Target molecules are directly captured from cellular lysates without the need for conventional RNA isolation. Polyadenylated mRNA is captured using magnetic beads, and gene-specific primers are simultaneously annealed to the captured target molecules in the first step of the process. After washing of the beads and buffer exchange, reverse transcription results in copies of first strand cDNA for specific molecular targets in the supernatant, which are then directly amplified by PCR. The PCR products are hybridized on a Ziplex instrument without purification after an exonuclease digestion, and the instrument automatically calculates expression levels from chemiluminescent signals. A copy of the complete poly-A+ transcriptome for each sample is retained covalently on the beads, which can be stored and used for additional analysis if desired.

We assessed the performance of the Landscape^+^ Breast Cancer assay using contrived samples and characterized the effects of WBC contamination on the assay’s performance. This assessment included spiking Parsortix harvests from HVs with breast cancer cell line cells as well as the comparison of assay results obtained from Parsortix harvests obtained from age and menopausal status matched HVs and MBC patients.

About 53 (75%) of the genes in the Landscape^+^ Breast Cancer assay responded to small amounts of RNA from the cell lines tested with an assay sensitivity adequate to potentially detect single cells. Differential responses between cell lines were observed for many of the genes, and the Principal Components Analysis with the 53 potentially responsive genes distinguished between the cell lines with amounts of RNA corresponding to single cell (20 pg). Hierarchical clustering also indicated that the cell line phenotypes were able to be distinguished when using as little as 20 pg of RNA.

In the presence of a 50-fold excess of WBC RNA (1 ng) relative to the amount of cell line RNA, genes with a background expression in WBCs were ineffective in distinguishing between 20 pg of RNA from different cell lines. However, a subset of the genes was able to distinguish the cell lines from each other as well as from unspiked WBC RNA, suggesting that specific genes can identify specific cell line phenotypes even in the presence of WBC background.

We further demonstrated that in the presence of varying levels of WBC contamination in Parsortix harvests from the blood of HVs, a subset of genes in the assay were differentially expressed in harvests spiked with a single cell of different cell lines and in harvests without a spiked cell. Although 100% of the tumor-derived cells present in a peripheral blood sample may not be captured by the Parsortix system in the microfluidic cassette and harvested, these results suggest that even a small number of tumor cells in a Parsortix harvest (down to a single CTC in a background of WBCs) can be detected and characterized with a panel of genes expressed in the tumor cells. The sensitivity demonstrated by the assay could provide a rapid and inexpensive method for characterizing individually picked cells from a Parsortix harvest to assess the heterogeneity of a CTC population. Similarly, specific gene panels targeted to the characteristics of the immune cell population potentially enriched in Parsortix harvests from cancer patient blood might also be informative.

Results obtained with the assay using aliquots of the Parsortix harvests from MBC patients and HVs showed that the expression of the PTPRC gene was highly variable between the samples and substantially greater on average in the MBC patients (Fig. 5**)**. This systematic difference in the presumed level of WBC contamination is likely to confound the relationship between the presence of tumor-derived CTCs and the expression level of genes that are expressed in WBCs. To minimize the effect of variable WBC contamination on the responses of the probes in the assay, the probes were ranked according to the slope between the response of each gene and the PTPRC gene. Genes with small or negative slopes are less likely to be influenced by the contaminating WBCs. The 10 genes at the top of the sorted gene list had essentially no responses above a threshold of 10 arbitrary units in any of the HV samples but had high levels of expression in some of the MBC samples. A signal level of 10 was arbitrarily chosen as a threshold above various sources of noise in the assay (including very low levels of expression apparent in WBCs for some genes). Different thresholds might be established for different genes, depending on the relative sensitivity of the primer/probe set and the relative expression levels of the genes in CTCs and WBCs.

Due to the heterogeneity of the tumor-derived CTCs in the MBC patients, few of the samples were positive above the threshold level for more than one of the 10 genes (Fig. 6). However, hierarchical clustering with these 10 genes resulted in a distinct cluster consisting of two thirds of the MBC patients without any of the HVs. One third of the MBC patients were in the other major cluster containing the HVs. Although the clinical significance is unclear, especially with such a small sample set, four of the five MBC samples in this cluster had PR-negative tumors (Additional file 9**)**. Recognizing that the 10 genes used in this classification may not be the optimal set of genes for the detection of CTCs in MBC patients, these results nevertheless suggest that similar studies with larger numbers of samples and perhaps with an expanded set of candidate genes may reveal a robust set of genes and a simple algorithm using the expression levels that could serve as a surrogate for (or complement to) conventional CTC enumeration. Analysis of parallel blood draws from the patients could also be used to correlate the presence of CTCs identified using immunofluorescent staining and with the gene expression profiles (26). Furthermore, the cell line results presented here indicate that gene expression analysis using the Landscape^+^ Breast Cancer assay may enable differentiation between cellular phenotypes in the samples that are likely to contain CTCs. Further study of this assay is warranted.

## Conclusions

The HyCEAD/Ziplex assay enabled the multiplex quantification of 72 genes with amounts of RNA corresponding to single cells. The resulting expression profiles unambiguously differentiated between different cell lines. The presence of WBC RNA or of RNA from Parsortix harvests from HV blood affected the differentiation between cell lines. However, with selection of genes with relatively low correlation with the number of contaminating WBCs (evaluated from the expression of the WBC marker PTPRC), cell lines were clearly differentiated from each other and from unspiked RNA. Some genes were found to differentiate between cell lines despite significant correlation with the expression of PTPRC.

HyCEAD/Ziplex analysis of Parsortix harvests from HVs and MBC patients indicated that the level of WBC contamination varied significantly between the HVs and MBC patients. To minimize the effect of the variable WBC contamination in the harvests, we selected a set of ten genes with little or no correlation with PTPRC and with expression greater in some MBC samples than in the HV samples. For all of these ten genes the expression levels were greater than in the HVs for only a small fraction of the MBC patients. Hierarchical clustering of the HV and MBC samples with these ten genes resulted in two main clusters, one of which consisted exclusively of MBC patients. The presence or absence of CTCs in the harvests is unknown, but we suggest that the MBC harvests that clustered with the HV samples may not have contained tumor-derived CTCs.

With careful selection of genes with relatively low expression in WBCs, the HyCEAD/Ziplex platform is an effective tool for molecular characterization of mRNA in small numbers of tumor cells harvested from blood.

## Supplementary Information

**Additional file 1:** Literature references for the 71 candidate genes.

**Additional file 2:** Landscape+ Breast Cancer assay primer and probe sequences. Sequences of Reverse Gene-Specificand Forward Gene-Specificprimers and Ziplex probes used in the Landscape+ Breast Cancer assay.

**Additional file 3:** Comparisons of three different primer/probe sets for genes in the breast cancer assay.

**Additional file 4:** Responses of breast cancer gene probes to increasing amounts of cell line total RNA from five cell lines.

**Additional file 5:** Hierarchical clustering of the data from 53 genes assayed with varying amounts of total RNA from five different cell lines with untransformed raw data for each heatmap.

**Additional file 6:** Responses of breast cancer gene probes to increasing amounts of cell line total RNA from five cell lines with WBC background.

**Additional file 7:** Hierarchical clustering of the data from 15 genes assayed with varying amounts of total RNA from five different cell lines with WBC background.

**Additional file 8:** Raw data for Figure 4.

**Additional file 9:** Clinical sample data.

**Additional file 10:** Signal intensities oif HVs and MBC patients.

**Additional file 11:** Raw data for Figure 7.

## Data Availability

All data generated or analyzed during this study are included in this published article.

## Abbreviations

CTCs: Circulating tumor cells
HyCEAD: Hybrid capture enrichment amplification and detection
MBC: Metastatic breast cancer
HVs: Healthy volunteers
qRT-PCR: Real-time quantitative PCR
PTPRC: Protein tyrosine phosphatase receptor type C
DPBS: Dulbecco’s phosphate buffered saline
DMEM: Dulbecco’s modified eagle medium
FBS: Fetal bovine serum
EMEM: Eagle’s minimum essential medium
ATCC: American type culture collection
EDTA: Ethylenediaminetetraacetic acid
ECACC: European collection of authenticated cell cultures
PBMCs: Human peripheral blood mononuclear cells
CMFDA: 5-Chloromethylfluorescein diacetate
FITC: Fluorescein isothiocyanate
TNBC: Triple-negative breast cancer
HER2: Human epidermal growth factor receptor 2
