# Supplementary material for: Novel method for highly multiplexed gene expression profiling of circulating tumor cells (CTCs) captured from the blood of women with metastatic breast cancer
Source: J Transl Med. 2023 Jun 26;21:414. doi: 10.1186/s12967-023-04242-z (PMC10291750; doi:10.1186/s12967-023-04242-z)
Supplement: Supplementary file 1 — Additional file 1: Literature references for the 71 candidate genes. [file 12967_2023_4242_MOESM1_ESM.pdf]

Additional File 1

Literature references for the 71 candidate genes

	Gene	Reference
1	AKT1	Lang, J. E., et al. (2018). "RNA-Seq of Circulating Tumor Cells in Stage II-III Breast Cancer." Ann Surg Oncol 25(8): 2261-2270.
2	AKT3	Lang, J. E., et al. (2018). "RNA-Seq of Circulating Tumor Cells in Stage II-III Breast Cancer." Ann Surg Oncol 25(8): 2261-2270.
3	ALDH1A1	Lang, J. E., et al. (2018). "RNA-Seq of Circulating Tumor Cells in Stage II-III Breast Cancer." Ann Surg Oncol 25(8): 2261-2270.
4	AR	Lang, J. E., et al. (2018). "RNA-Seq of Circulating Tumor Cells in Stage II-III Breast Cancer." Ann Surg Oncol 25(8): 2261-2270.
5	ATM	Lang, J. E., et al. (2018). "RNA-Seq of Circulating Tumor Cells in Stage II-III Breast Cancer." Ann Surg Oncol 25(8): 2261-2270.
6	BARD1	Lang, J. E., et al. (2018). "RNA-Seq of Circulating Tumor Cells in Stage II-III Breast Cancer." Ann Surg Oncol 25(8): 2261-2270.
7	BRAF	Lang, J. E., et al. (2018). "RNA-Seq of Circulating Tumor Cells in Stage II-III Breast Cancer." Ann Surg Oncol 25(8): 2261-2270.
8	BRCA1	Lang, J. E., et al. (2018). "RNA-Seq of Circulating Tumor Cells in Stage II-III Breast Cancer." Ann Surg Oncol 25(8): 2261-2270.
9	BRCA2	Lang, J. E., et al. (2018). "RNA-Seq of Circulating Tumor Cells in Stage II-III Breast Cancer." Ann Surg Oncol 25(8): 2261-2270.
10	CCND1	Lang, J. E., et al. (2018). "RNA-Seq of Circulating Tumor Cells in Stage II-III Breast Cancer." Ann Surg Oncol 25(8): 2261-2270.
11	CCND2	Lang, J. E., et al. (2018). "RNA-Seq of Circulating Tumor Cells in Stage II-III Breast Cancer." Ann Surg Oncol 25(8): 2261-2270.
12	CCND3	Lang, J. E., et al. (2018). "RNA-Seq of Circulating Tumor Cells in Stage II-III Breast Cancer." Ann Surg Oncol 25(8): 2261-2270.
13	CCNE1	Lang, J. E., et al. (2018). "RNA-Seq of Circulating Tumor Cells in Stage II-III Breast Cancer." Ann Surg Oncol 25(8): 2261-2270.
14	CD274	Kaur, P., et al. (2020). "A Pilot Study for the Feasibility of Exome-Sequencing in Circulating Tumor Cells Versus Single Metastatic Biopsies in Breast Cancer." Int J Mol Sci 21(14).
15	CD3D	Ring A, Porras TA, Campo D, et al. The whole transcriptional landscape of circulating tumor cells compared to metastases in stage IV breast cancer [poster abstract]. Presented at the San Antonio Breast Cancer Symposium, December 2017.
16	CDH1	Lang, J. E., et al. (2018). "RNA-Seq of Circulating Tumor Cells in Stage II-III Breast Cancer." Ann Surg Oncol 25(8): 2261-2270.
17	CDK4	Lang, J. E., et al. (2018). "RNA-Seq of Circulating Tumor Cells in Stage II-III Breast Cancer." Ann Surg Oncol 25(8): 2261-2270.
18	CDK6	Lang, J. E., et al. (2018). "RNA-Seq of Circulating Tumor Cells in Stage II-III Breast Cancer." Ann Surg Oncol 25(8): 2261-2270.
19	CDKN2A	Lang, J. E., et al. (2018). "RNA-Seq of Circulating Tumor Cells in Stage II-III Breast Cancer." Ann Surg Oncol 25(8): 2261-2270.
20	CXCL9	Lang, J. E., et al. (2018). "RNA-Seq of Circulating Tumor Cells in Stage II-III Breast Cancer." Ann Surg Oncol 25(8): 2261-2270.
21	CXXC5	Lang, J. E., et al. (2018). "RNA-Seq of Circulating Tumor Cells in Stage II-III Breast Cancer." Ann Surg Oncol 25(8): 2261-2270.
22	EGFR	Lang, J. E., et al. (2018). "RNA-Seq of Circulating Tumor Cells in Stage II-III Breast Cancer." Ann Surg Oncol 25(8): 2261-2270.
23	EPCAM	Lang, J. E., et al. (2018). "RNA-Seq of Circulating Tumor Cells in Stage II-III Breast Cancer." Ann Surg Oncol 25(8): 2261-2270.
24	ERBB2	Porras, T. B., et al. (2018). "Challenges in using liquid biopsies for gene expression profiling." Oncotarget 9(6): 7036-7053.
25	ERBB3	Sieuwerts, A. M., et al. (2011). "mRNA and microRNA expression profiles in circulating tumor cells and primary tumors of metastatic breast cancer patients." Clin Cancer Res 17(11): 3600-3618.
26	ESR1	Lang, J. E., et al. (2018). "RNA-Seq of Circulating Tumor Cells in Stage II-III Breast Cancer." Ann Surg Oncol 25(8): 2261-2270.
27	ESR2	Lang, J. E., et al. (2018). "RNA-Seq of Circulating Tumor Cells in Stage II-III Breast Cancer." Ann Surg Oncol 25(8): 2261-2270.
28	FBXW7	Lang, J. E., et al. (2018). "RNA-Seq of Circulating Tumor Cells in Stage II-III Breast Cancer." Ann Surg Oncol 25(8): 2261-2270.

29	FGF1	Lang, J. E., et al. (2018). "RNA-Seq of Circulating Tumor Cells in Stage II-III Breast Cancer." Ann Surg Oncol 25(8): 2261-2270.
30	FGFR1	Kaur, P., et al. (2020). "A Pilot Study for the Feasibility of Exome-Sequencing in Circulating Tumor Cells Versus Single Metastatic Biopsies in Breast Cancer." Int J Mol Sci 21(14).
31	FGFR2	Lang, J. E., et al. (2018). "RNA-Seq of Circulating Tumor Cells in Stage II-III Breast Cancer." Ann Surg Oncol 25(8): 2261-2270.
32	FGFR3	Sieuwerts, A. M., et al. (2011). "mRNA and microRNA expression profiles in circulating tumor cells and primary tumors of metastatic breast cancer patients." Clin Cancer Res 17(11): 3600-3618.
33	FGFR4	Porras, T. B., et al. (2018). "Challenges in using liquid biopsies for gene expression profiling." Oncotarget 9(6): 7036-7053.
34	FOXA1	Porras, T. B., et al. (2018). "Challenges in using liquid biopsies for gene expression profiling." Oncotarget 9(6): 7036-7053.
35	IGF1	Kaur, P., et al. (2020). "A Pilot Study for the Feasibility of Exome-Sequencing in Circulating Tumor Cells Versus Single Metastatic Biopsies in Breast Cancer." Int J Mol Sci 21(14).
36	IL12B	Thomas, H., et al. (2014). "Interaction with mesenchymal stem cells provokes natural killer cells for enhanced IL-12/IL-18-induced interferon-gamma secretion." Mediators Inflamm 2014: 143463.
37	IL15	Lang, J. E., et al. (2018). "RNA-Seq of Circulating Tumor Cells in Stage II-III Breast Cancer." Ann Surg Oncol 25(8): 2261-2270.
38	IL23A	Lang, J. E., et al. (2018). "RNA-Seq of Circulating Tumor Cells in Stage II-III Breast Cancer." Ann Surg Oncol 25(8): 2261-2270.
39	IL4	Lang, J. E., et al. (2018). "RNA-Seq of Circulating Tumor Cells in Stage II-III Breast Cancer." Ann Surg Oncol 25(8): 2261-2270.
40	IL6	Lang, J. E., et al. (2018). "RNA-Seq of Circulating Tumor Cells in Stage II-III Breast Cancer." Ann Surg Oncol 25(8): 2261-2270.
41	JAK2	Lang, J. E., et al. (2018). "RNA-Seq of Circulating Tumor Cells in Stage II-III Breast Cancer." Ann Surg Oncol 25(8): 2261-2270.
42	KRAS	Lang, J. E., et al. (2018). "RNA-Seq of Circulating Tumor Cells in Stage II-III Breast Cancer." Ann Surg Oncol 25(8): 2261-2270.
43	KRT14	Porras, T. B., et al. (2018). "Challenges in using liquid biopsies for gene expression profiling." Oncotarget 9(6): 7036-7053.
44	KRT17	Porras, T. B., et al. (2018). "Challenges in using liquid biopsies for gene expression profiling." Oncotarget 9(6): 7036-7053.
45	KRT19	Lang, J. E., et al. (2018). "RNA-Seq of Circulating Tumor Cells in Stage II-III Breast Cancer." Ann Surg Oncol 25(8): 2261-2270.
46	MAP2K1	Lang, J. E., et al. (2018). "RNA-Seq of Circulating Tumor Cells in Stage II-III Breast Cancer." Ann Surg Oncol 25(8): 2261-2270.
47	MAP2K7	Marino, N., et al. (2014). "Identification and validation of genes with expression patterns inverse to multiple metastasis suppressor genes in breast cancer cell lines." Clin Exp Metastasis 31(7): 771-786.
48	MAP3K1	Lang, J. E., et al. (2018). "RNA-Seq of Circulating Tumor Cells in Stage II-III Breast Cancer." Ann Surg Oncol 25(8): 2261-2270.
49	MET	Lang, J. E., et al. (2018). "RNA-Seq of Circulating Tumor Cells in Stage II-III Breast Cancer." Ann Surg Oncol 25(8): 2261-2270.
50	MKI67	Porras, T. B., et al. (2018). "Challenges in using liquid biopsies for gene expression profiling." Oncotarget 9(6): 7036-7053.
51	MTOR	Ring A, Porras TA, Campo D, et al. The whole transcriptional landscape of circulating tumor cells compared to metastases in stage IV breast cancer [poster abstract]. Presented at the San Antonio Breast Cancer Symposium, December 2017.
52	MYC	Lang, J. E., et al. (2018). "RNA-Seq of Circulating Tumor Cells in Stage II-III Breast Cancer." Ann Surg Oncol 25(8): 2261-2270.
53	NANOG	Lang, J. E., et al. (2018). "RNA-Seq of Circulating Tumor Cells in Stage II-III Breast Cancer." Ann Surg Oncol 25(8): 2261-2270.
54	NF1	Lang, J. E., et al. (2018). "RNA-Seq of Circulating Tumor Cells in Stage II-III Breast Cancer." Ann Surg Oncol 25(8): 2261-2270.
55	NOTCH1	Lang, J. E., et al. (2018). "RNA-Seq of Circulating Tumor Cells in Stage II-III Breast Cancer." Ann Surg Oncol 25(8): 2261-2270.
56	NOTCH4	Lang, J. E., et al. (2018). "RNA-Seq of Circulating Tumor Cells in Stage II-III Breast Cancer." Ann Surg Oncol 25(8): 2261-2270.

57	PALB2	Lang, J. E., et al. (2018). "RNA-Seq of Circulating Tumor Cells in Stage II-III Breast Cancer." Ann Surg Oncol 25(8): 2261-2270.
58	PGR	Lang, J. E., et al. (2018). "RNA-Seq of Circulating Tumor Cells in Stage II-III Breast Cancer." Ann Surg Oncol 25(8): 2261-2270.
59	PIK3CA	Lang, J. E., et al. (2018). "RNA-Seq of Circulating Tumor Cells in Stage II-III Breast Cancer." Ann Surg Oncol 25(8): 2261-2270.
60	PROM1	Lang, J. E., et al. (2018). "RNA-Seq of Circulating Tumor Cells in Stage II-III Breast Cancer." Ann Surg Oncol 25(8): 2261-2270.
61	PTTG1	Lang, J. E., et al. (2018). "RNA-Seq of Circulating Tumor Cells in Stage II-III Breast Cancer." Ann Surg Oncol 25(8): 2261-2270.
62	RB1	Lang, J. E., et al. (2018). "RNA-Seq of Circulating Tumor Cells in Stage II-III Breast Cancer." Ann Surg Oncol 25(8): 2261-2270.
63	RPTOR	Lang, J. E., et al. (2018). "RNA-Seq of Circulating Tumor Cells in Stage II-III Breast Cancer." Ann Surg Oncol 25(8): 2261-2270.
64	SFRP1	Lang, J. E., et al. (2018). "RNA-Seq of Circulating Tumor Cells in Stage II-III Breast Cancer." Ann Surg Oncol 25(8): 2261-2270.
65	SPARC	Magbanua, M. J. M., et al. (2018). "Expanded Genomic Profiling of Circulating Tumor Cells in Metastatic Breast Cancer Patients to Assess Biomarker Status and Biology Over Time (CALGB 40502 and CALGB 40503, Alliance)." Clin Cancer Res 24(6): 1486-1499.
66	TBX3	Lang, J. E., et al. (2018). "RNA-Seq of Circulating Tumor Cells in Stage II-III Breast Cancer." Ann Surg Oncol 25(8): 2261-2270.
67	TWIST1	Lang, J. E., et al. (2018). "RNA-Seq of Circulating Tumor Cells in Stage II-III Breast Cancer." Ann Surg Oncol 25(8): 2261-2270.
68	WNT1	Lang, J. E., et al. (2018). "RNA-Seq of Circulating Tumor Cells in Stage II-III Breast Cancer." Ann Surg Oncol 25(8): 2261-2270.
69	WNT5A	Reinholz, M. M., et al. (2010). Expression profiling of formalin-fixed paraffin-embedded primary breast tumors using cancer-specific and whole genome gene panels on the DASL(R) platform. BMC Med Genomics. England. 3: 60.
70	WNT5B	Fina, E. (2022). "Signatures of Breast Cancer Progression in the Blood: What Could Be Learned from Circulating Tumor Cell Transcriptomes." Cancers (Basel) 14(22).
71	WNT8A	Jiang, S., et al. (2019). "WNT5B governs the phenotype of basal-like breast cancer by activating WNT signaling." Cell Commun Signal 17(1): 109.
