# Supplementary material for: Novel method for highly multiplexed gene expression profiling of circulating tumor cells (CTCs) captured from the blood of women with metastatic breast cancer
Source: J Transl Med. 2023 Jun 26;21:414. doi: 10.1186/s12967-023-04242-z (PMC10291750; doi:10.1186/s12967-023-04242-z)
Supplement: Supplementary file 3 — Additional file 3: Comparisons of three different primer/probe sets for genes in the breast cancer assay. [file 12967_2023_4242_MOESM3_ESM.pdf]

Additonal File 3
Comparisons of three different primer/probe sets for genes in the breast cancer assay.

Pages 2 and 3 - Responses of three primer/probe designs for each of six genes originally designed for an ovarian cancer assay. Each primer/probe set was screened against four cell lines, white blood cell RNA and UHR RNA. The solid line indicates equality between the responses of the two primer/probe sets compared in each log-log plot. The dotted line is a linear regression line. "R" is the correlatioin coefficient, and "X indicates the approximate fold-difference in the response of the two primer/probe sets.

Pages 5 to 19 - Responses of three primer/probe designs for each of 59 genes designed for the breast cancer assay. Each primer/probe set was screened against twelve cell lines, white blood cell RNA (1,000 and 5,000 pg) and UHR RNA. The solid line indicates equality between the responses of the two primer/probe sets compared in each log-log plot. The dotted lines indicate saturation levels of the Ziplex instrument response. The symbol legend for the samples is on page 4.

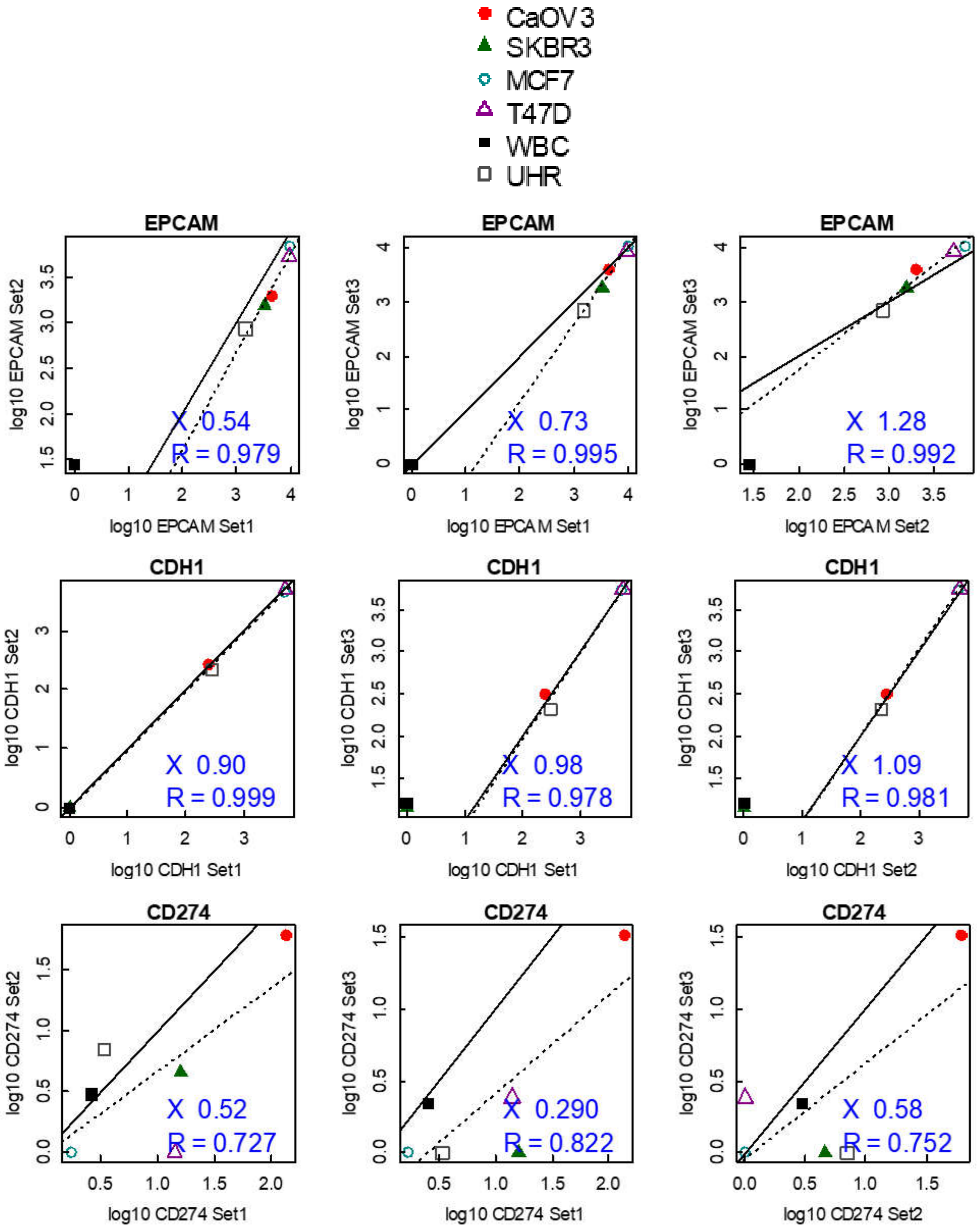
CaOV3
SKBR3
MCF7
T47D
WBC
UHR
EPCAM
log10 EPCAM Set2
log10 EPCAM Set1
X 0.54
R = 0.979
EPCAM
log10 EPCAM Set3
log10 EPCAM Set1
X 0.73
R = 0.995
EPCAM
log10 EPCAM Set3
log10 EPCAM Set2
X 1.28
R = 0.992
CDH1
log10 CDH1 Set2
log10 CDH1 Set1
X 0.90
R = 0.999
CDH1
log10 CDH1 Set3
log10 CDH1 Set1
X 0.98
R = 0.978
CDH1
log10 CDH1 Set3
log10 CDH1 Set2
X 1.09
R = 0.981
CD274
log10 CD274 Set2
log10 CD274 Set1
X 0.52
R = 0.727
CD274
log10 CD274 Set3
log10 CD274 Set1
X 0.290
R = 0.822
CD274
log10 CD274 Set3
log10 CD274 Set2
X 0.58
R = 0.752

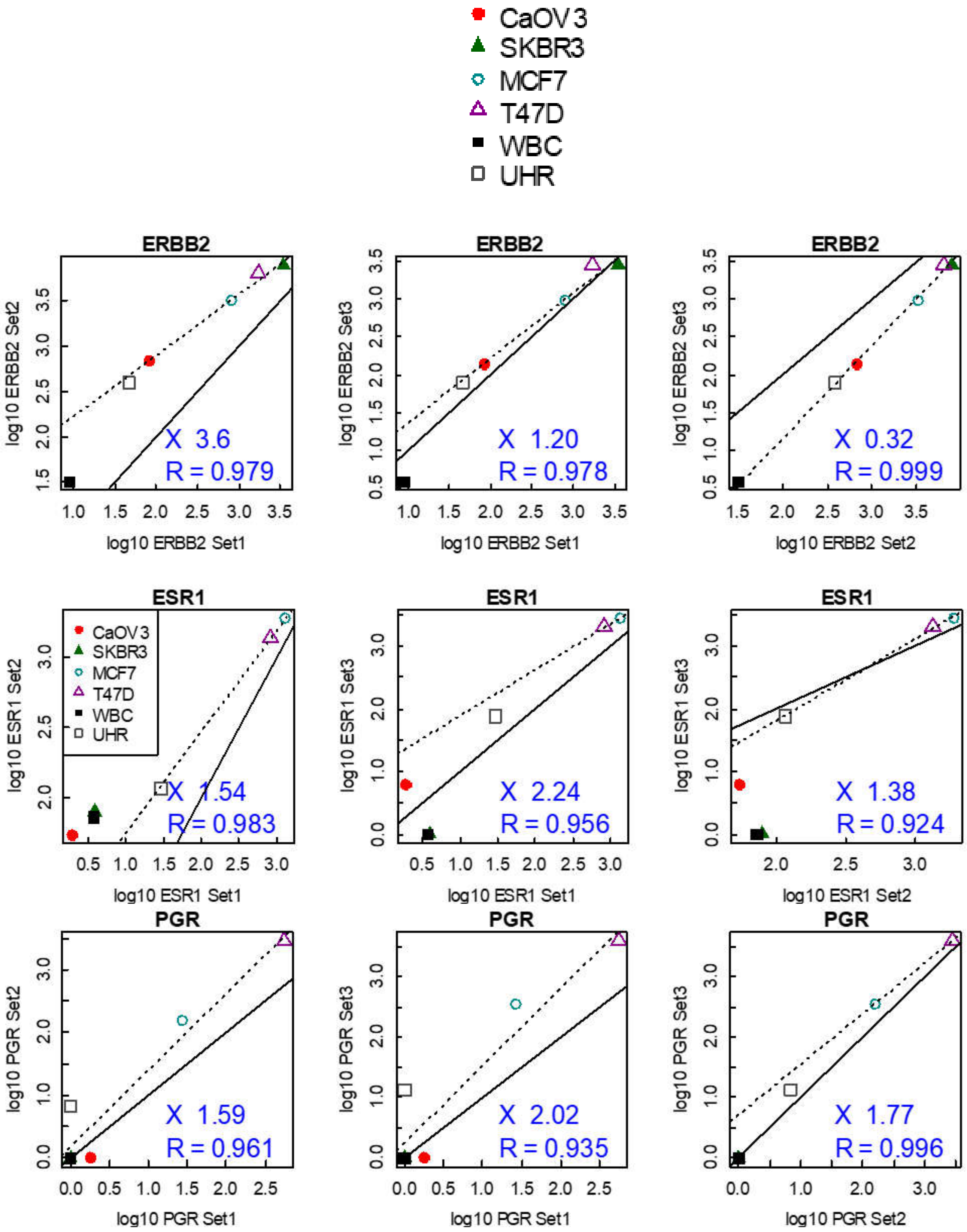
CaOV3
SKBR3
MCF7
T47D
WBC
UHR
ERBB2
log10 ERBB2 Set2
log10 ERBB2 Set1
X 3.6
R = 0.979
ERBB2
log10 ERBB2 Set3
log10 ERBB2 Set1
X 1.20
R = 0.978
ERBB2
log10 ERBB2 Set3
log10 ERBB2 Set2
X 0.32
R = 0.999
ESR1
log10 ESR1 Set2
log10 ESR1 Set1
X 1.54
R = 0.983
ESR1
log10 ESR1 Set3
log10 ESR1 Set1
X 2.24
R = 0.956
ESR1
log10 ESR1 Set3
log10 ESR1 Set2
X 1.38
R = 0.924
PGR
log10 PGR Set2
log10 PGR Set1
X 1.59
R = 0.961
PGR
log10 PGR Set3
log10 PGR Set1
X 2.02
R = 0.935
PGR
log10 PGR Set3
log10 PGR Set2
X 1.77
R = 0.996

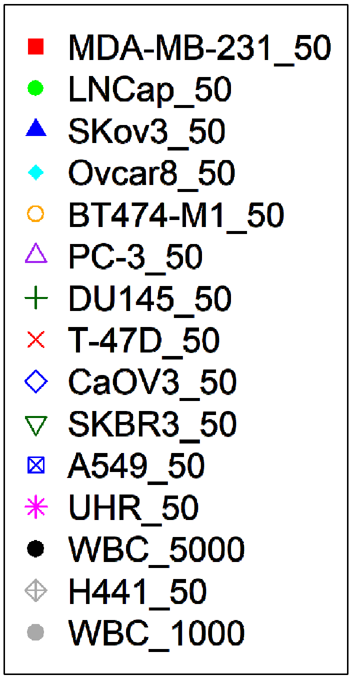
MDA-MB-231_50
LNCap_50
SKov3_50
Ovcar8_50
BT474-M1_50
PC-3_50
DU145_50
T-47D_50
CaOV3_50
SKBR3_50
A549_50
UHR_50
WBC_5000
H441_50
WBC_1000

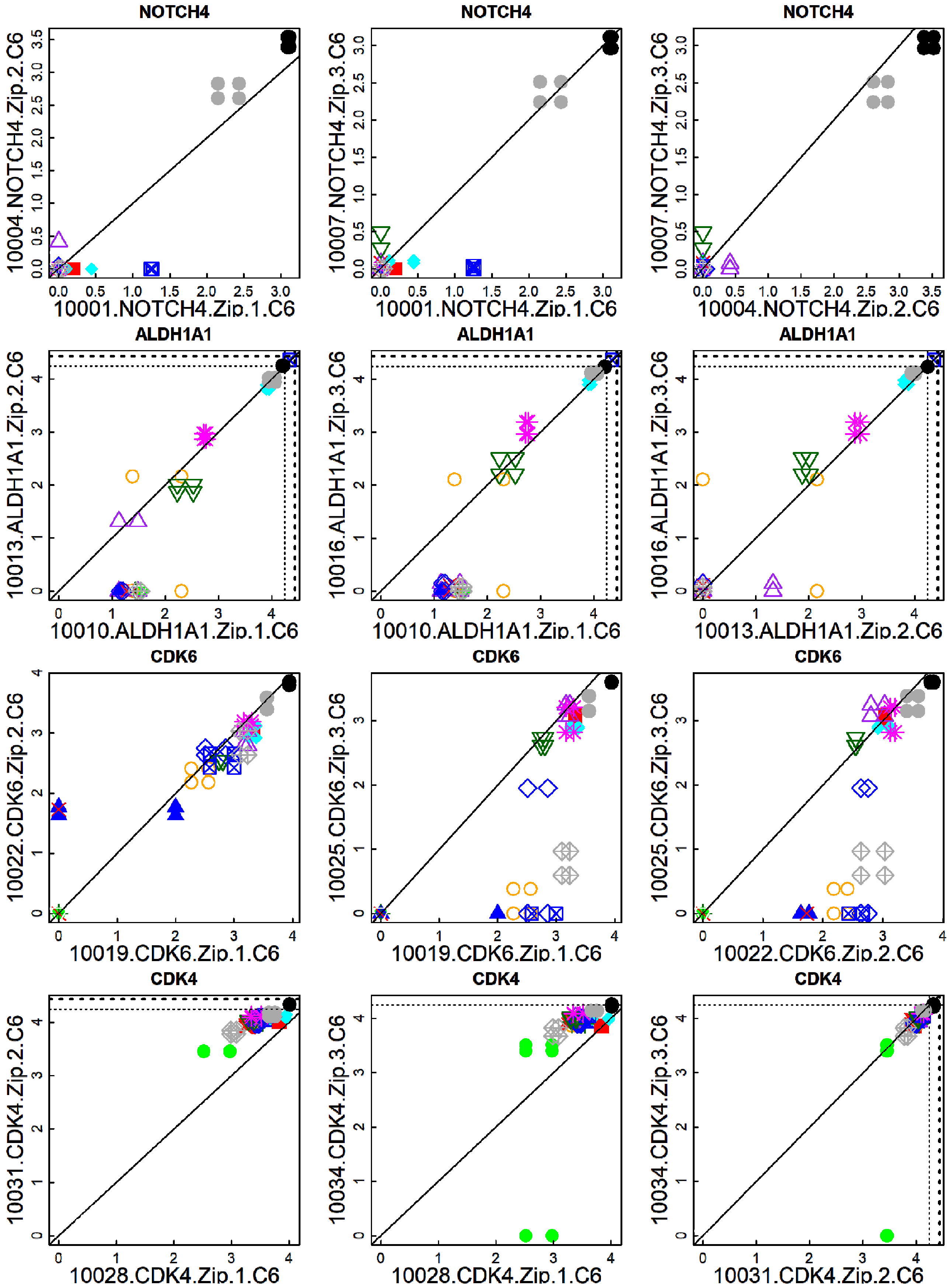
NOTCH4
10004.NOTCH4.Zip.2.C6
10001.NOTCH4.Zip.1.C6
NOTCH4
10007.NOTCH4.Zip.3.C6
10001.NOTCH4.Zip.1.C6
NOTCH4
10007.NOTCH4.Zip.3.C6
10004.NOTCH4.Zip.2.C6
ALDH1A1
10013.ALDH1A1.Zip.2.C6
10010.ALDH1A1.Zip.1.C6
ALDH1A1
10016.ALDH1A1.Zip.3.C6
10010.ALDH1A1.Zip.1.C6
ALDH1A1
10016.ALDH1A1.Zip.3.C6
10013.ALDH1A1.Zip.2.C6
CDK6
10022.CDK6.Zip.2.C6
10019.CDK6.Zip.1.C6
CDK6
10025.CDK6.Zip.3.C6
10019.CDK6.Zip.1.C6
CDK6
10025.CDK6.Zip.3.C6
10022.CDK6.Zip.2.C6
CDK4
10031.CDK4.Zip.2.C6
10028.CDK4.Zip.1.C6
CDK4
10034.CDK4.Zip.3.C6
10028.CDK4.Zip.1.C6
CDK4
10034.CDK4.Zip.3.C6
10031.CDK4.Zip.2.C6

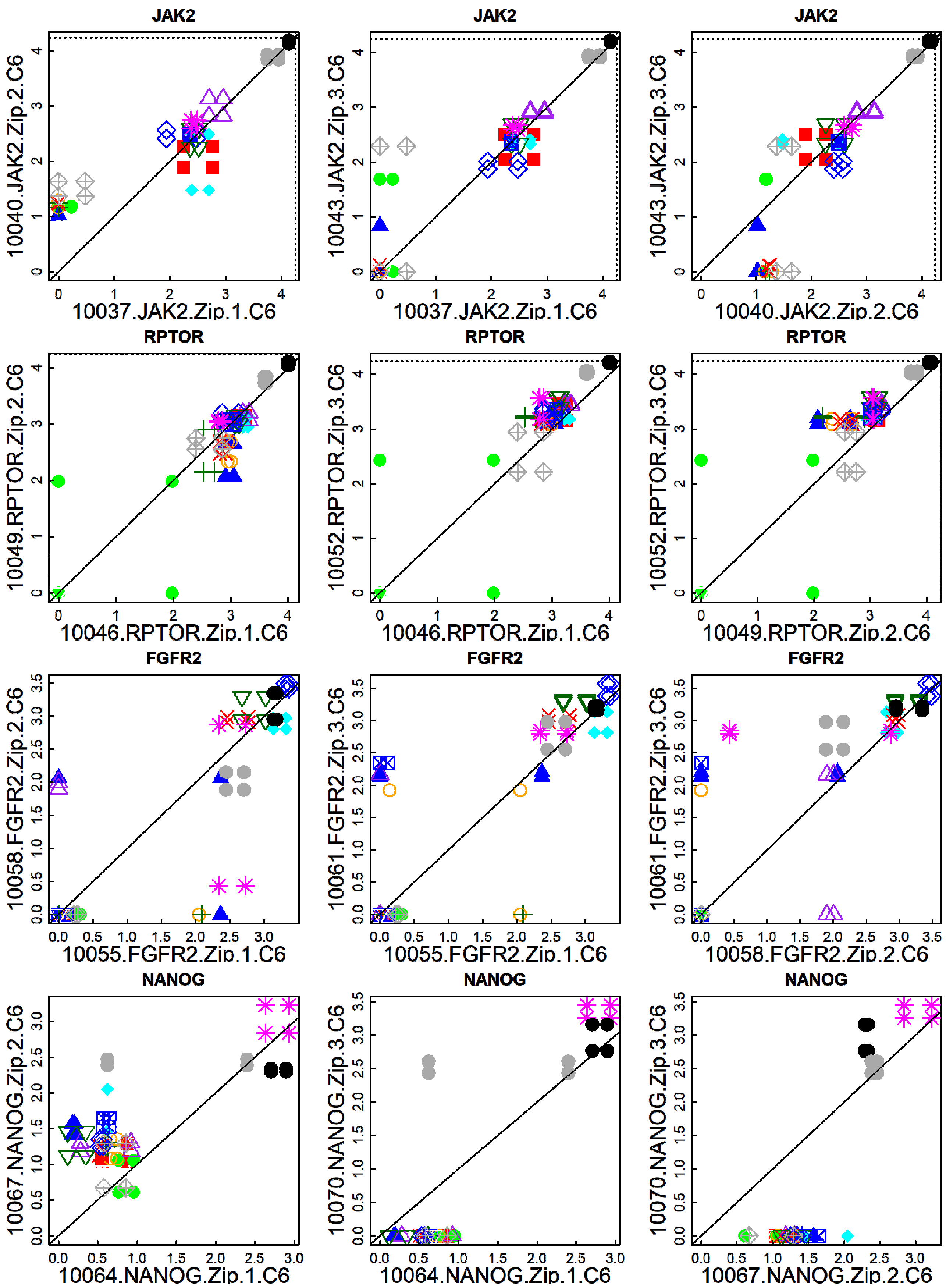
JAK2
10040.JAK2.Zip.2.C6
10037.JAK2.Zip.1.C6
JAK2
10043.JAK2.Zip.3.C6
10037.JAK2.Zip.1.C6
JAK2
10043.JAK2.Zip.3.C6
10040.JAK2.Zip.2.C6
RPTOR
10049.RPTOR.Zip.2.C6
10046.RPTOR.Zip.1.C6
RPTOR
10052.RPTOR.Zip.3.C6
10046.RPTOR.Zip.1.C6
RPTOR
10052.RPTOR.Zip.3.C6
10049.RPTOR.Zip.2.C6
FGFR2
10058.FGFR2.Zip.2.C6
10055.FGFR2.Zip.1.C6
FGFR2
10061.FGFR2.Zip.3.C6
10055.FGFR2.Zip.1.C6
FGFR2
10061.FGFR2.Zip.3.C6
10058.FGFR2.Zip.2.C6
NANOG
10067.NANOG.Zip.2.C6
10064.NANOG.Zip.1.C6
NANOG
10070.NANOG.Zip.3.C6
10064.NANOG.Zip.1.C6
NANOG
10070.NANOG.Zip.3.C6
10067.NANOG.Zip.2.C6

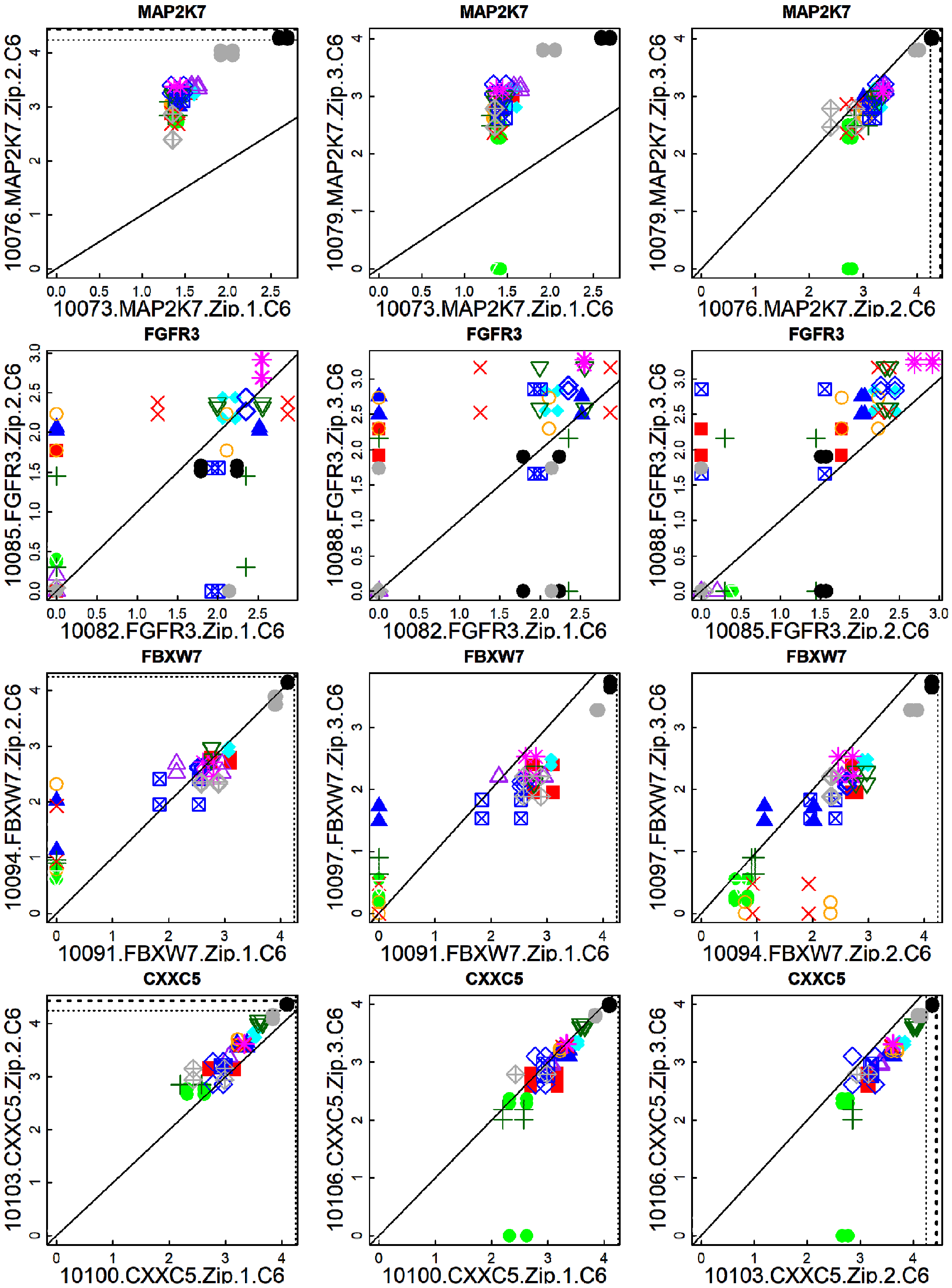
MAP2K7
10076.MAP2K7.Zip.2.C6
10073.MAP2K7.Zip.1.C6
MAP2K7
10079.MAP2K7.Zip.3.C6
10073.MAP2K7.Zip.1.C6
MAP2K7
10079.MAP2K7.Zip.3.C6
10076.MAP2K7.Zip.2.C6
FGFR3
10085.FGFR3.Zip.2.C6
10082.FGFR3.Zip.1.C6
FGFR3
10088.FGFR3.Zip.3.C6
10082.FGFR3.Zip.1.C6
FGFR3
10088.FGFR3.Zip.3.C6
10085.FGFR3.Zip.2.C6
FBXW7
10094.FBXW7.Zip.2.C6
10091.FBXW7.Zip.1.C6
FBXW7
10097.FBXW7.Zip.3.C6
10091.FBXW7.Zip.1.C6
FBXW7
10097.FBXW7.Zip.3.C6
10094.FBXW7.Zip.2.C6
CXXC5
10103.CXXC5.Zip.2.C6
10100.CXXC5.Zip.1.C6
CXXC5
10106.CXXC5.Zip.3.C6
10100.CXXC5.Zip.1.C6
CXXC5
10106.CXXC5.Zip.3.C6
10103.CXXC5.Zip.2.C6

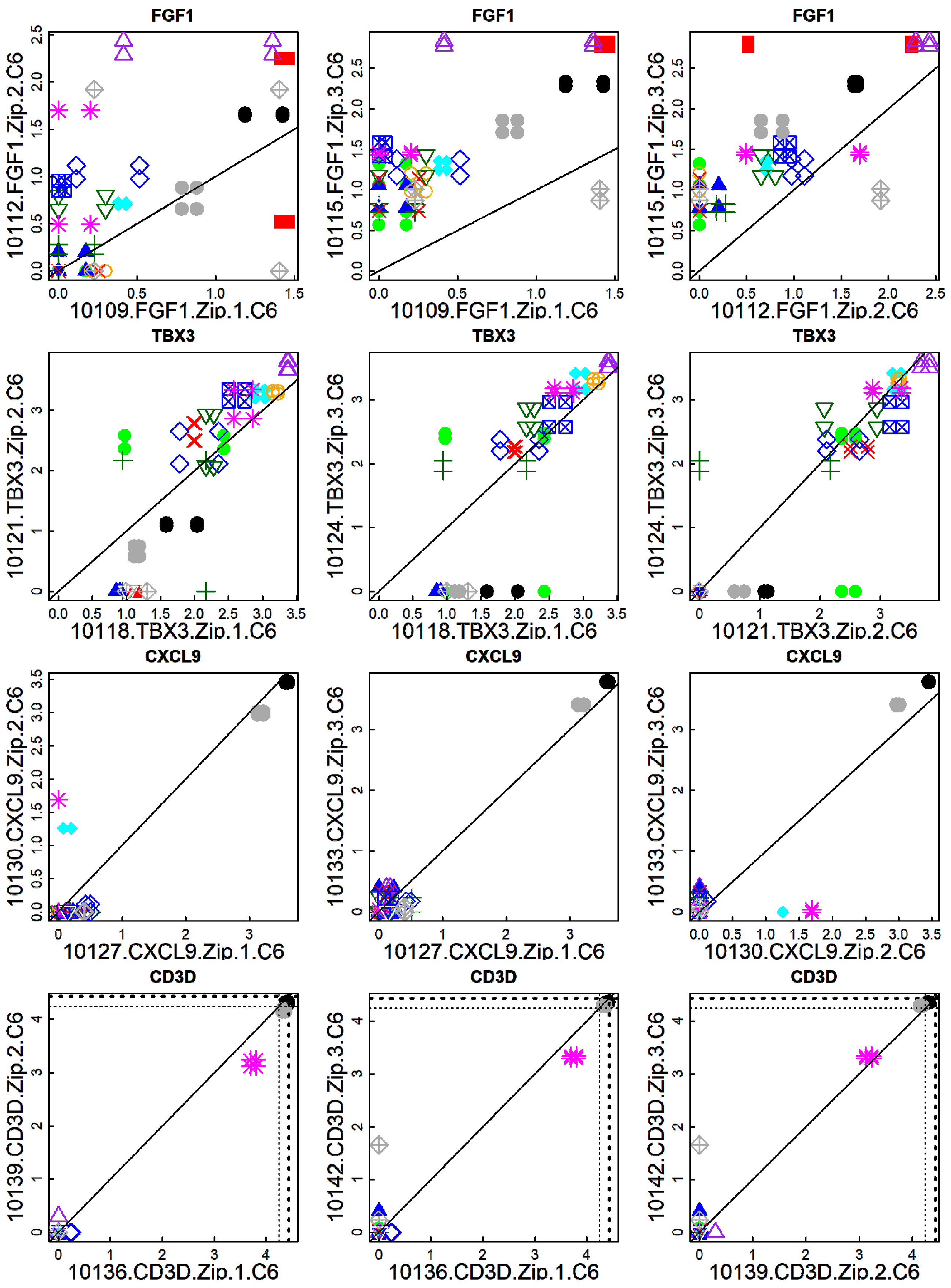
FGF1
10112.FGF1.Zip.2.C6
10109.FGF1.Zip.1.C6
FGF1
10115.FGF1.Zip.3.C6
10109.FGF1.Zip.1.C6
FGF1
10115.FGF1.Zip.3.C6
10112.FGF1.Zip.2.C6
TBX3
10121.TBX3.Zip.2.C6
10118.TBX3.Zip.1.C6
TBX3
10124.TBX3.Zip.3.C6
10118.TBX3.Zip.1.C6
TBX3
10124.TBX3.Zip.3.C6
10121.TBX3.Zip.2.C6
CXCL9
10130.CXCL9.Zip.2.C6
10127.CXCL9.Zip.1.C6
CXCL9
10133.CXCL9.Zip.3.C6
10127.CXCL9.Zip.1.C6
CXCL9
10133.CXCL9.Zip.3.C6
10130.CXCL9.Zip.2.C6
CD3D
10139.CD3D.Zip.2.C6
10136.CD3D.Zip.1.C6
CD3D
10142.CD3D.Zip.3.C6
10136.CD3D.Zip.1.C6
CD3D
10142.CD3D.Zip.3.C6
10139.CD3D.Zip.2.C6

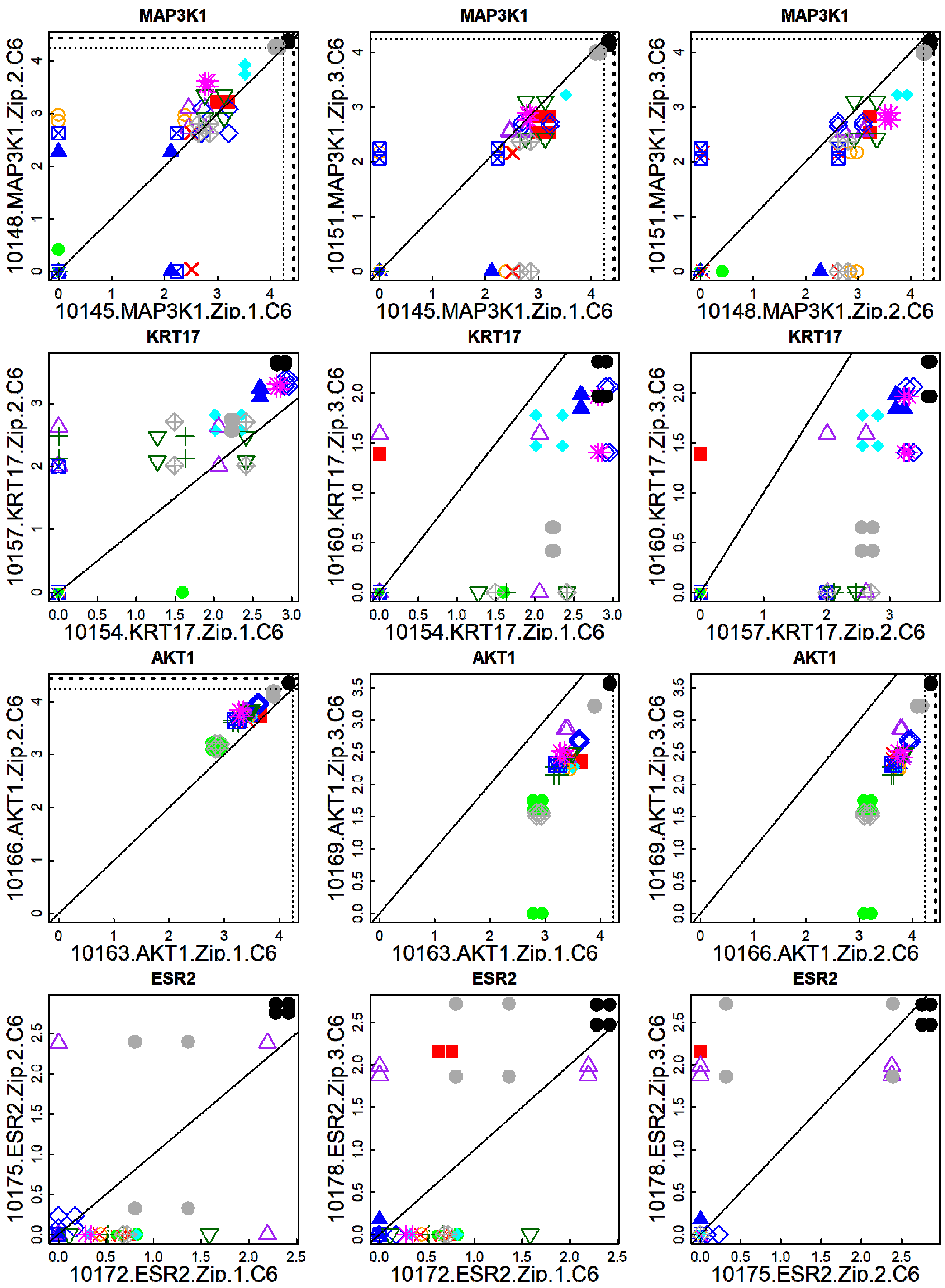
MAP3K1
10148.MAP3K1.Zip.2.C6
10145.MAP3K1.Zip.1.C6
MAP3K1
10151.MAP3K1.Zip.3.C6
10145.MAP3K1.Zip.1.C6
MAP3K1
10151.MAP3K1.Zip.3.C6
10148.MAP3K1.Zip.2.C6
KRT17
10157.KRT17.Zip.2.C6
10154.KRT17.Zip.1.C6
KRT17
10160.KRT17.Zip.3.C6
10154.KRT17.Zip.1.C6
KRT17
10160.KRT17.Zip.3.C6
10157.KRT17.Zip.2.C6
AKT1
10166.AKT1.Zip.2.C6
10163.AKT1.Zip.1.C6
AKT1
10169.AKT1.Zip.3.C6
10163.AKT1.Zip.1.C6
AKT1
10169.AKT1.Zip.3.C6
10166.AKT1.Zip.2.C6
ESR2
10175.ESR2.Zip.2.C6
10172.ESR2.Zip.1.C6
ESR2
10178.ESR2.Zip.3.C6
10172.ESR2.Zip.1.C6
ESR2
10178.ESR2.Zip.3.C6
10175.ESR2.Zip.2.C6

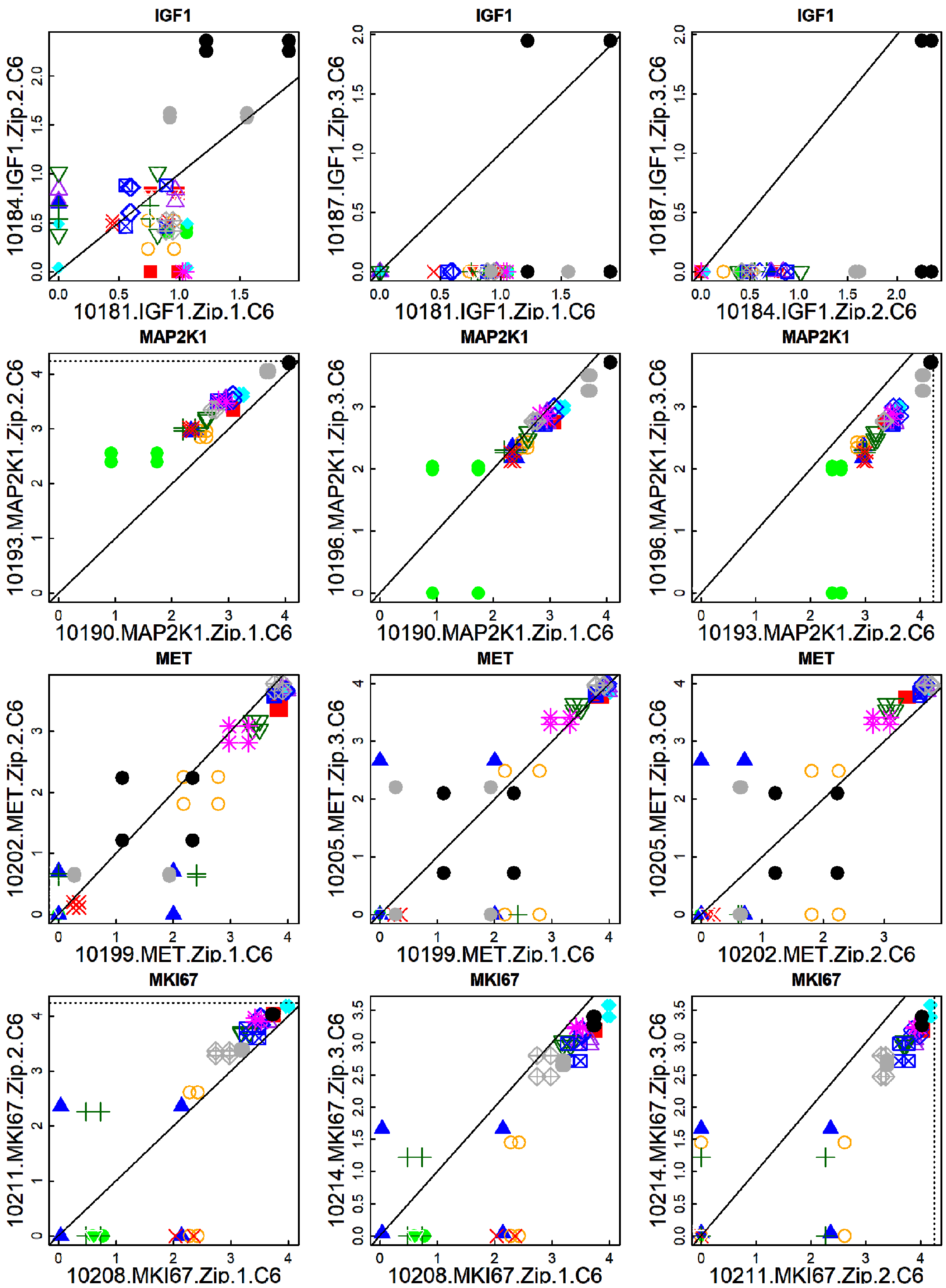
IGF1
10184.IGF1.Zip.2.C6
10181.IGF1.Zip.1.C6
IGF1
10187.IGF1.Zip.3.C6
10181.IGF1.Zip.1.C6
IGF1
10187.IGF1.Zip.3.C6
10184.IGF1.Zip.2.C6
MAP2K1
10193.MAP2K1.Zip.2.C6
10190.MAP2K1.Zip.1.C6
MAP2K1
10196.MAP2K1.Zip.3.C6
10190.MAP2K1.Zip.1.C6
MAP2K1
10196.MAP2K1.Zip.3.C6
10193.MAP2K1.Zip.2.C6
MET
10202.MET.Zip.2.C6
10199.MET.Zip.1.C6
MET
10205.MET.Zip.3.C6
10199.MET.Zip.1.C6
MET
10205.MET.Zip.3.C6
10202.MET.Zip.2.C6
MKI67
10211.MKI67.Zip.2.C6
10208.MKI67.Zip.1.C6
MKI67
10214.MKI67.Zip.3.C6
10208.MKI67.Zip.1.C6
MKI67
10214.MKI67.Zip.3.C6
10211.MKI67.Zip.2.C6

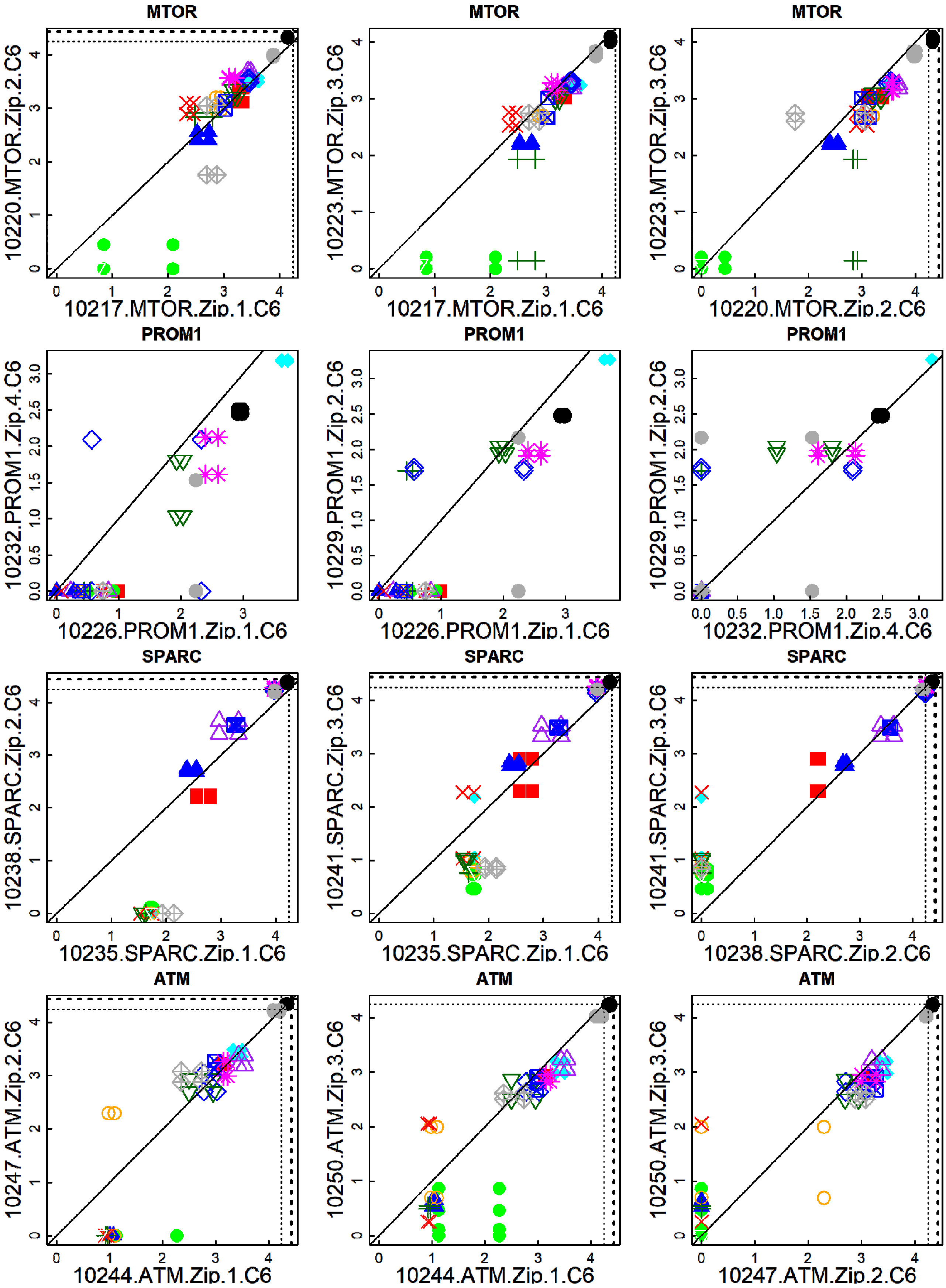
MTOR
10220.MTOR.Zip.2.C6
10217.MTOR.Zip.1.C6
MTOR
10223.MTOR.Zip.3.C6
10217.MTOR.Zip.1.C6
MTOR
10223.MTOR.Zip.3.C6
10220.MTOR.Zip.2.C6
PROM1
10232.PROM1.Zip.4.C6
10226.PROM1.Zip.1.C6
PROM1
10229.PROM1.Zip.2.C6
10226.PROM1.Zip.1.C6
PROM1
10229.PROM1.Zip.2.C6
10232.PROM1.Zip.4.C6
SPARC
10238.SPARC.Zip.2.C6
10235.SPARC.Zip.1.C6
SPARC
10241.SPARC.Zip.3.C6
10235.SPARC.Zip.1.C6
SPARC
10241.SPARC.Zip.3.C6
10238.SPARC.Zip.2.C6
ATM
10247.ATM.Zip.2.C6
10244.ATM.Zip.1.C6
ATM
10250.ATM.Zip.3.C6
10244.ATM.Zip.1.C6
ATM
10250.ATM.Zip.3.C6
10247.ATM.Zip.2.C6

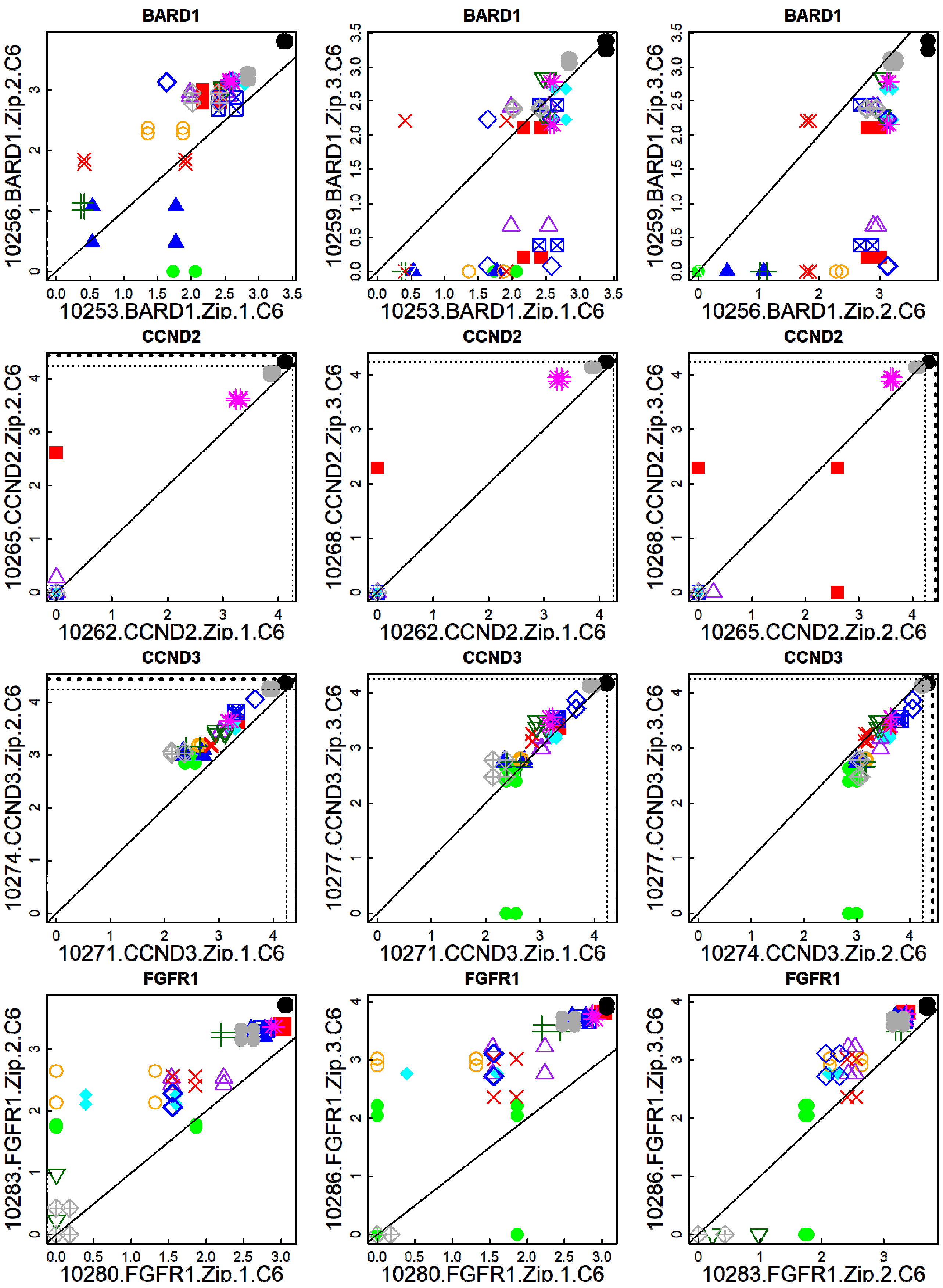
BARD1
10256.BARD1.Zip.2.C6
10253.BARD1.Zip.1.C6
BARD1
10259.BARD1.Zip.3.C6
10253.BARD1.Zip.1.C6
BARD1
10259.BARD1.Zip.3.C6
10256.BARD1.Zip.2.C6
CCND2
10265.CCND2.Zip.2.C6
10262.CCND2.Zip.1.C6
CCND2
10268.CCND2.Zip.3.C6
10262.CCND2.Zip.1.C6
CCND2
10268.CCND2.Zip.3.C6
10265.CCND2.Zip.2.C6
CCND3
10274.CCND3.Zip.2.C6
10271.CCND3.Zip.1.C6
CCND3
10277.CCND3.Zip.3.C6
10271.CCND3.Zip.1.C6
CCND3
10277.CCND3.Zip.3.C6
10274.CCND3.Zip.2.C6
FGFR1
10283.FGFR1.Zip.2.C6
10280.FGFR1.Zip.1.C6
FGFR1
10286.FGFR1.Zip.3.C6
10280.FGFR1.Zip.1.C6
FGFR1
10286.FGFR1.Zip.3.C6
10283.FGFR1.Zip.2.C6

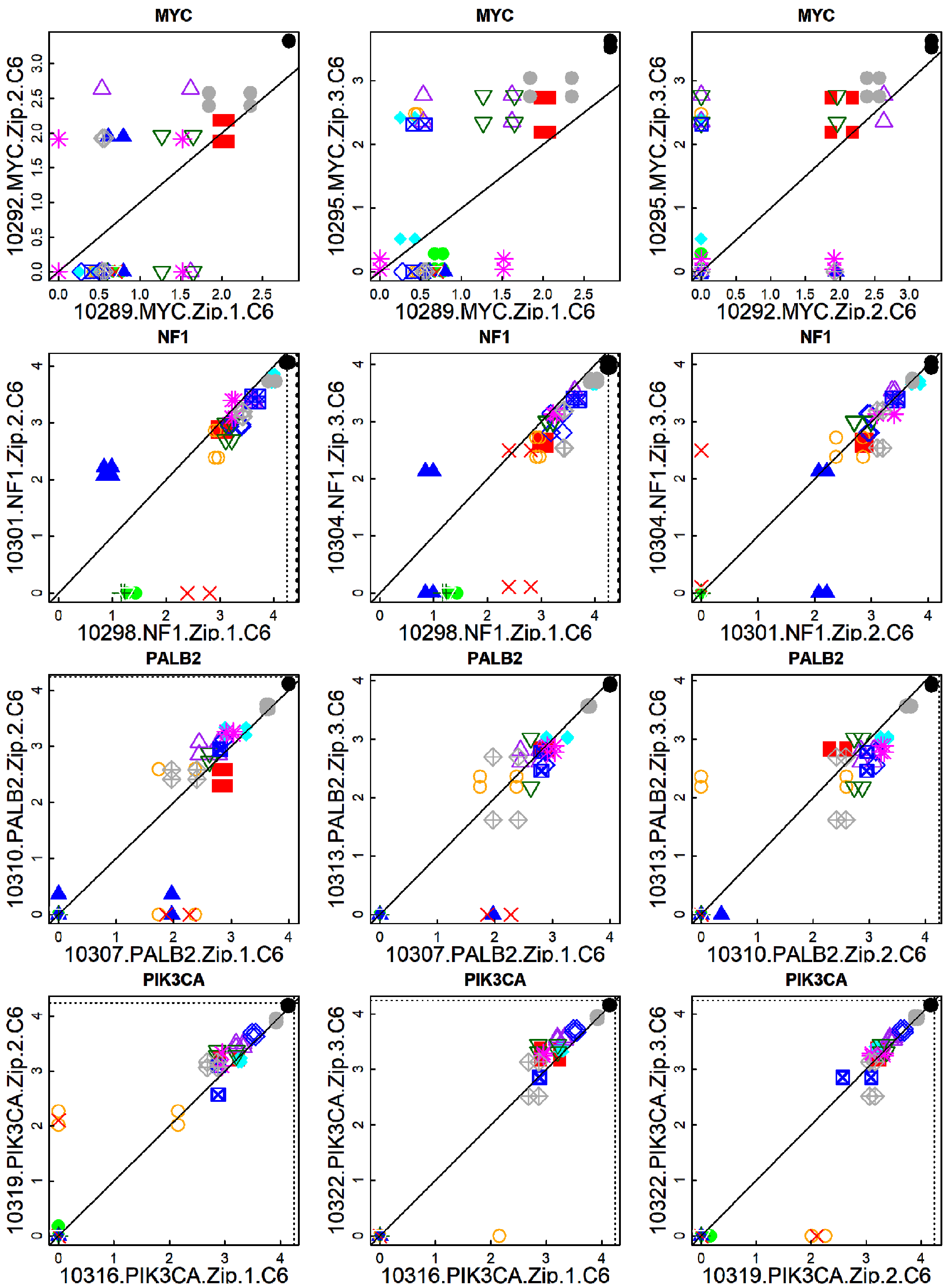
MYC
10292.MYC.Zip.2.C6
10289.MYC.Zip.1.C6
MYC
10295.MYC.Zip.3.C6
10289.MYC.Zip.1.C6
MYC
10295.MYC.Zip.3.C6
10292.MYC.Zip.2.C6
NF1
10301.NF1.Zip.2.C6
10298.NF1.Zip.1.C6
NF1
10304.NF1.Zip.3.C6
10298.NF1.Zip.1.C6
NF1
10304.NF1.Zip.3.C6
10301.NF1.Zip.2.C6
PALB2
10310.PALB2.Zip.2.C6
10307.PALB2.Zip.1.C6
PALB2
10313.PALB2.Zip.3.C6
10307.PALB2.Zip.1.C6
PALB2
10313.PALB2.Zip.3.C6
10310.PALB2.Zip.2.C6
PIK3CA
10319.PIK3CA.Zip.2.C6
10316.PIK3CA.Zip.1.C6
PIK3CA
10322.PIK3CA.Zip.3.C6
10316.PIK3CA.Zip.1.C6
PIK3CA
10322.PIK3CA.Zip.3.C6
10319.PIK3CA.Zip.2.C6

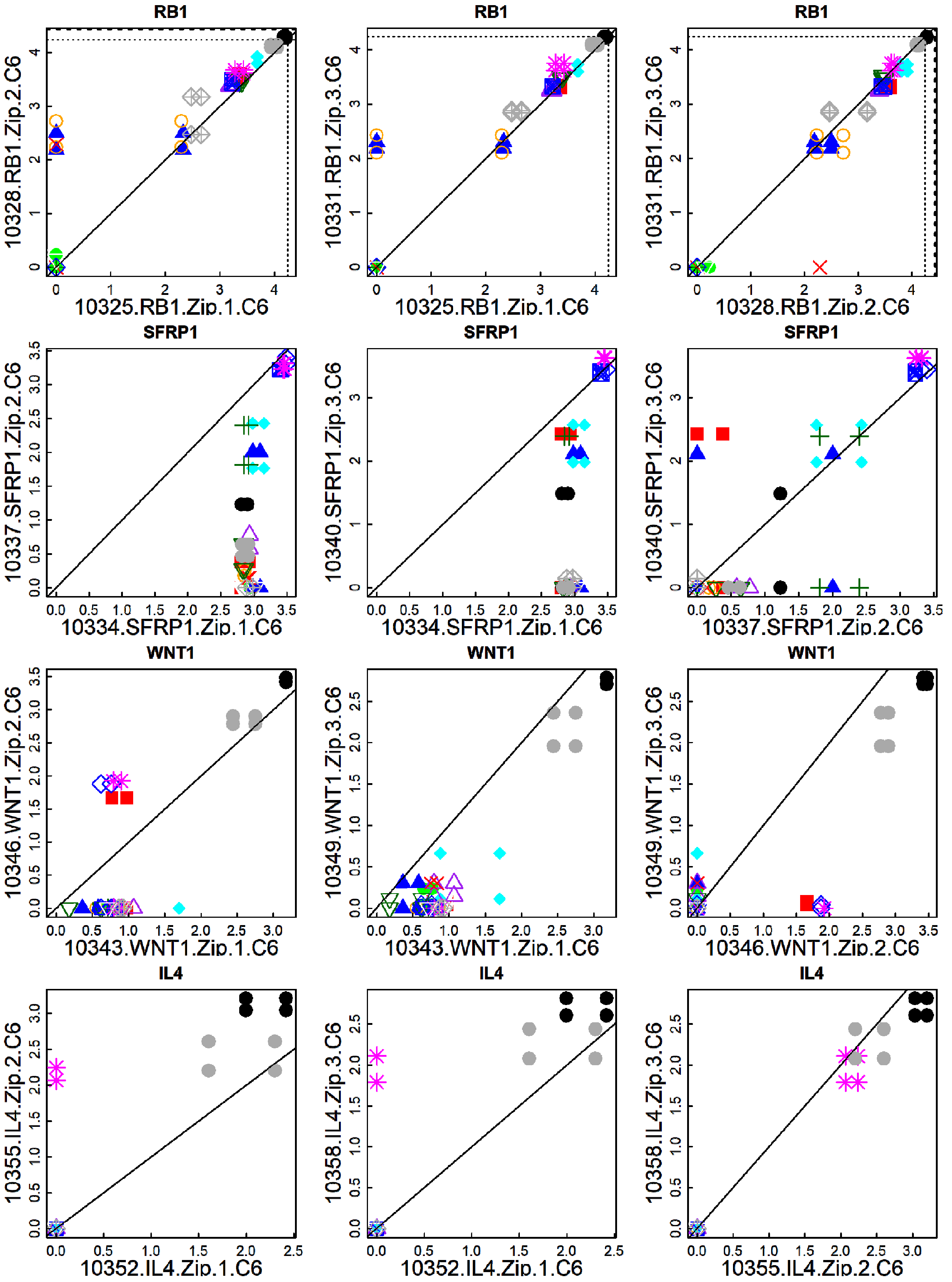
RB1
10328.RB1.Zip.2.C6
10325.RB1.Zip.1.C6
RB1
10331.RB1.Zip.3.C6
10325.RB1.Zip.1.C6
RB1
10331.RB1.Zip.3.C6
10328.RB1.Zip.2.C6
SFRP1
10337.SFRP1.Zip.2.C6
10334.SFRP1.Zip.1.C6
SFRP1
10340.SFRP1.Zip.3.C6
10334.SFRP1.Zip.1.C6
SFRP1
10340.SFRP1.Zip.3.C6
10337.SFRP1.Zip.2.C6
WNT1
10346.WNT1.Zip.2.C6
10343.WNT1.Zip.1.C6
WNT1
10349.WNT1.Zip.3.C6
10343.WNT1.Zip.1.C6
WNT1
10349.WNT1.Zip.3.C6
10346.WNT1.Zip.2.C6
IL4
10355.IL4.Zip.2.C6
10352.IL4.Zip.1.C6
IL4
10358.IL4.Zip.3.C6
10352.IL4.Zip.1.C6
IL4
10358.IL4.Zip.3.C6
10355.IL4.Zip.2.C6

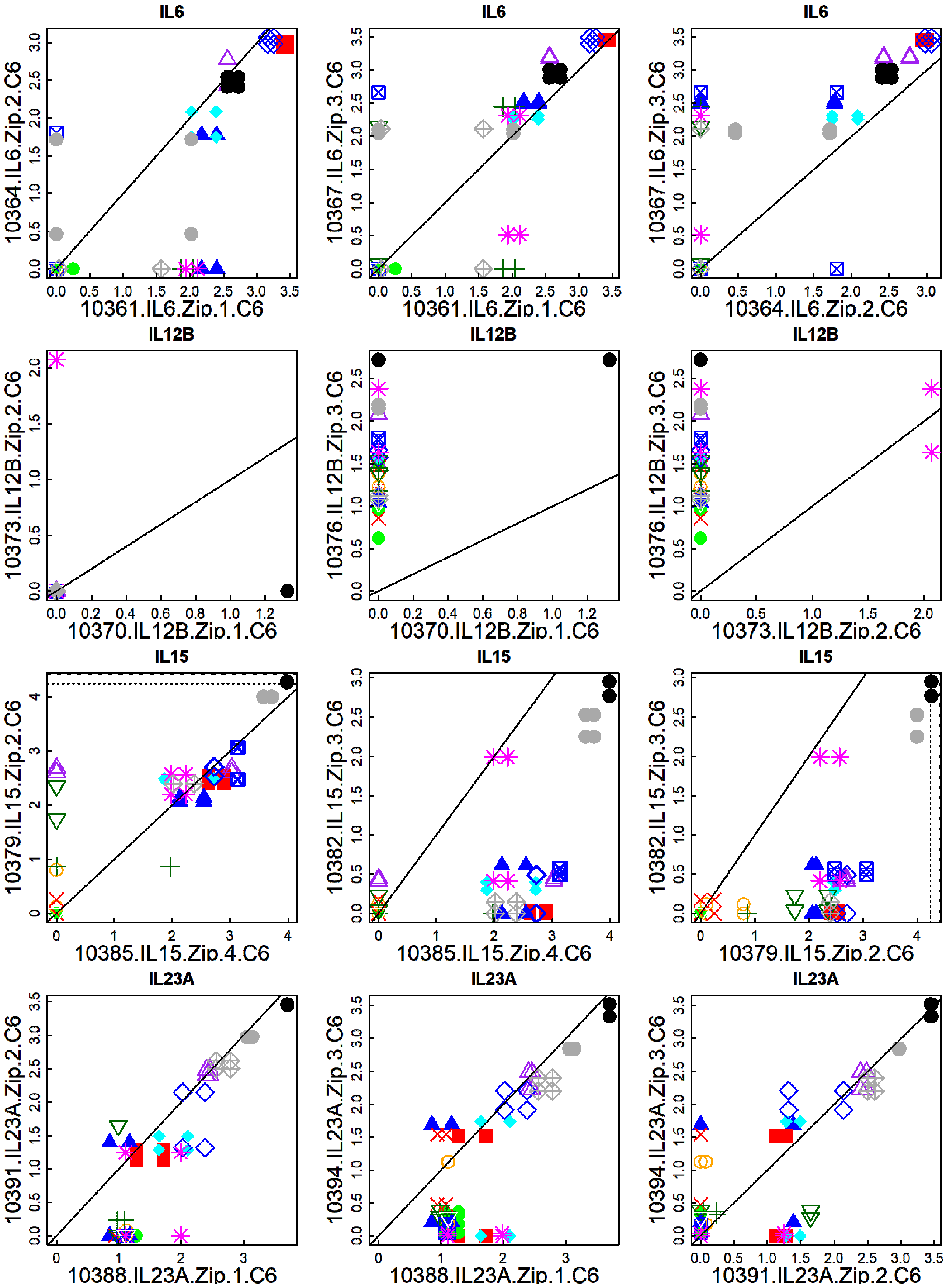
IL6
10361.IL6.Zip.1.C6
10364.IL6.Zip.2.C6
IL6
10361.IL6.Zip.1.C6
10367.IL6.Zip.3.C6
IL6
10364.IL6.Zip.2.C6
10367.IL6.Zip.3.C6
IL12B
10370.IL12B.Zip.1.C6
10373.IL12B.Zip.2.C6
IL12B
10370.IL12B.Zip.1.C6
10376.IL12B.Zip.3.C6
IL12B
10373.IL12B.Zip.2.C6
10376.IL12B.Zip.3.C6
IL15
10385.IL15.Zip.4.C6
10379.IL15.Zip.2.C6
IL15
10385.IL15.Zip.4.C6
10382.IL15.Zip.3.C6
IL15
10379.IL15.Zip.2.C6
10382.IL15.Zip.3.C6
IL23A
10388.IL23A.Zip.1.C6
10391.IL23A.Zip.2.C6
IL23A
10388.IL23A.Zip.1.C6
10394.IL23A.Zip.3.C6
IL23A
10391.IL23A.Zip.2.C6
10394.IL23A.Zip.3.C6

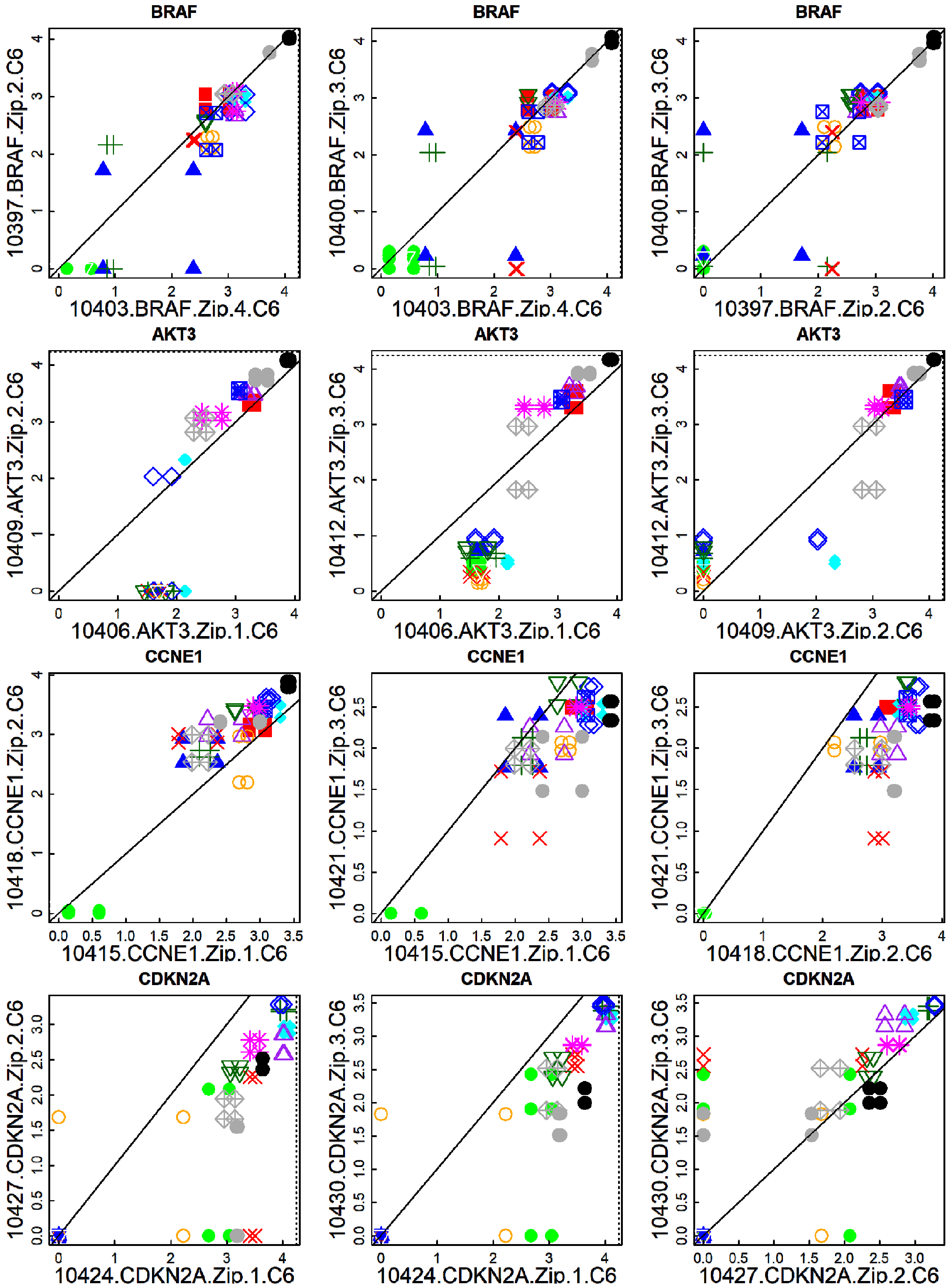
BRAF
10397.BRAF.Zip.2.C6
10403.BRAF.Zip.4.C6
BRAF
10400.BRAF.Zip.3.C6
10403.BRAF.Zip.4.C6
BRAF
10400.BRAF.Zip.3.C6
10397.BRAF.Zip.2.C6
AKT3
10409.AKT3.Zip.2.C6
10406.AKT3.Zip.1.C6
AKT3
10412.AKT3.Zip.3.C6
10406.AKT3.Zip.1.C6
AKT3
10412.AKT3.Zip.3.C6
10409.AKT3.Zip.2.C6
CCNE1
10418.CCNE1.Zip.2.C6
10415.CCNE1.Zip.1.C6
CCNE1
10421.CCNE1.Zip.3.C6
10415.CCNE1.Zip.1.C6
CCNE1
10421.CCNE1.Zip.3.C6
10418.CCNE1.Zip.2.C6
CDKN2A
10427.CDKN2A.Zip.2.C6
10424.CDKN2A.Zip.1.C6
CDKN2A
10430.CDKN2A.Zip.3.C6
10424.CDKN2A.Zip.1.C6
CDKN2A
10430.CDKN2A.Zip.3.C6
10427.CDKN2A.Zip.2.C6

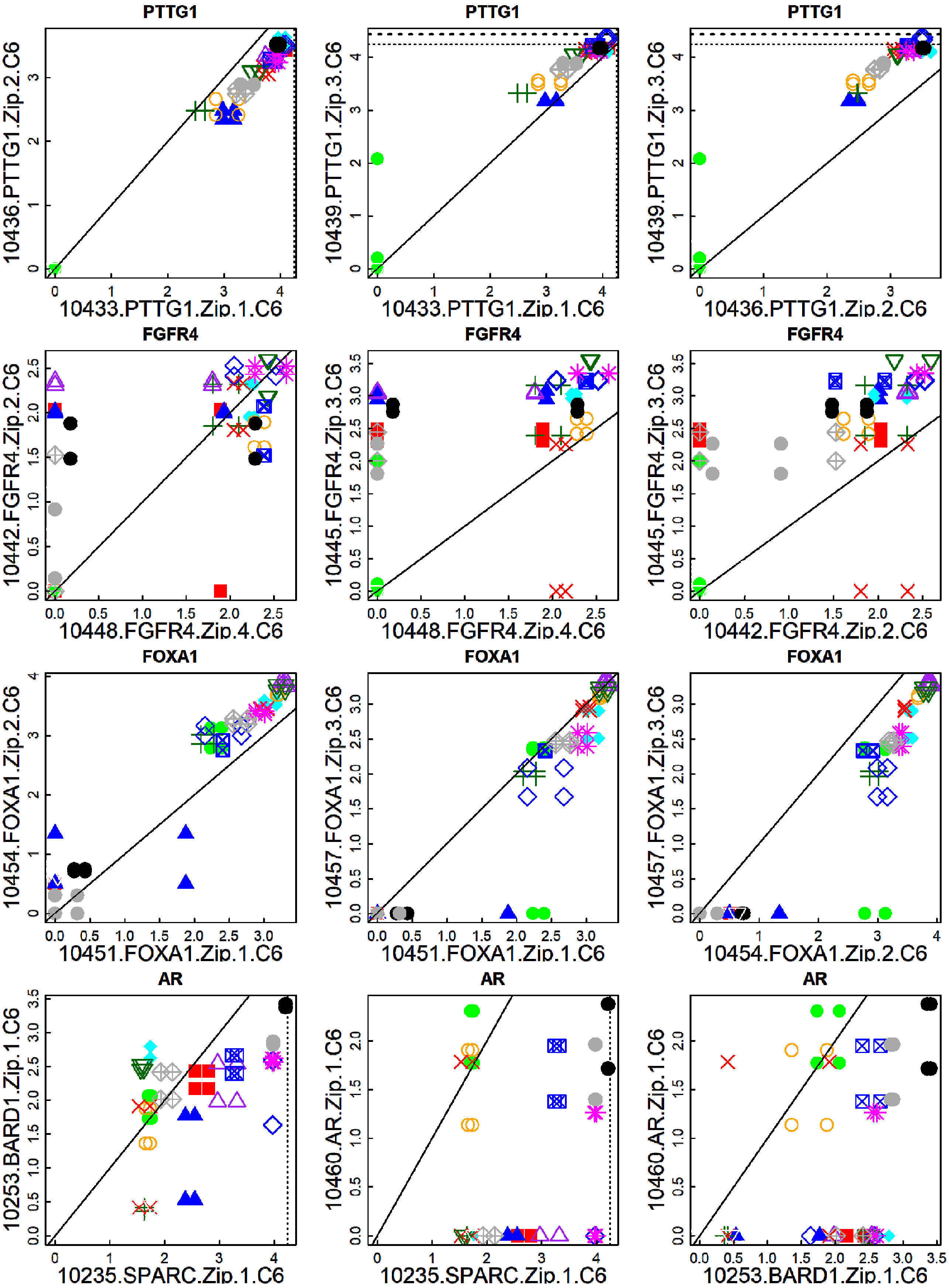
PTTG1
10436.PTTG1.Zip.2.C6
10433.PTTG1.Zip.1.C6
PTTG1
10439.PTTG1.Zip.3.C6
10433.PTTG1.Zip.1.C6
PTTG1
10439.PTTG1.Zip.3.C6
10436.PTTG1.Zip.2.C6
FGFR4
10442.FGFR4.Zip.2.C6
10448.FGFR4.Zip.4.C6
FGFR4
10445.FGFR4.Zip.3.C6
10448.FGFR4.Zip.4.C6
FGFR4
10445.FGFR4.Zip.3.C6
10442.FGFR4.Zip.2.C6
FOXA1
10454.FOXA1.Zip.2.C6
10451.FOXA1.Zip.1.C6
FOXA1
10457.FOXA1.Zip.3.C6
10451.FOXA1.Zip.1.C6
FOXA1
10457.FOXA1.Zip.3.C6
10454.FOXA1.Zip.2.C6
AR
10253.BARD1.Zip.1.C6
10235.SPARC.Zip.1.C6
AR
10460.AR.Zip.1.C6
10235.SPARC.Zip.1.C6
AR
10460.AR.Zip.1.C6
10253.BARD1.Zip.1.C6

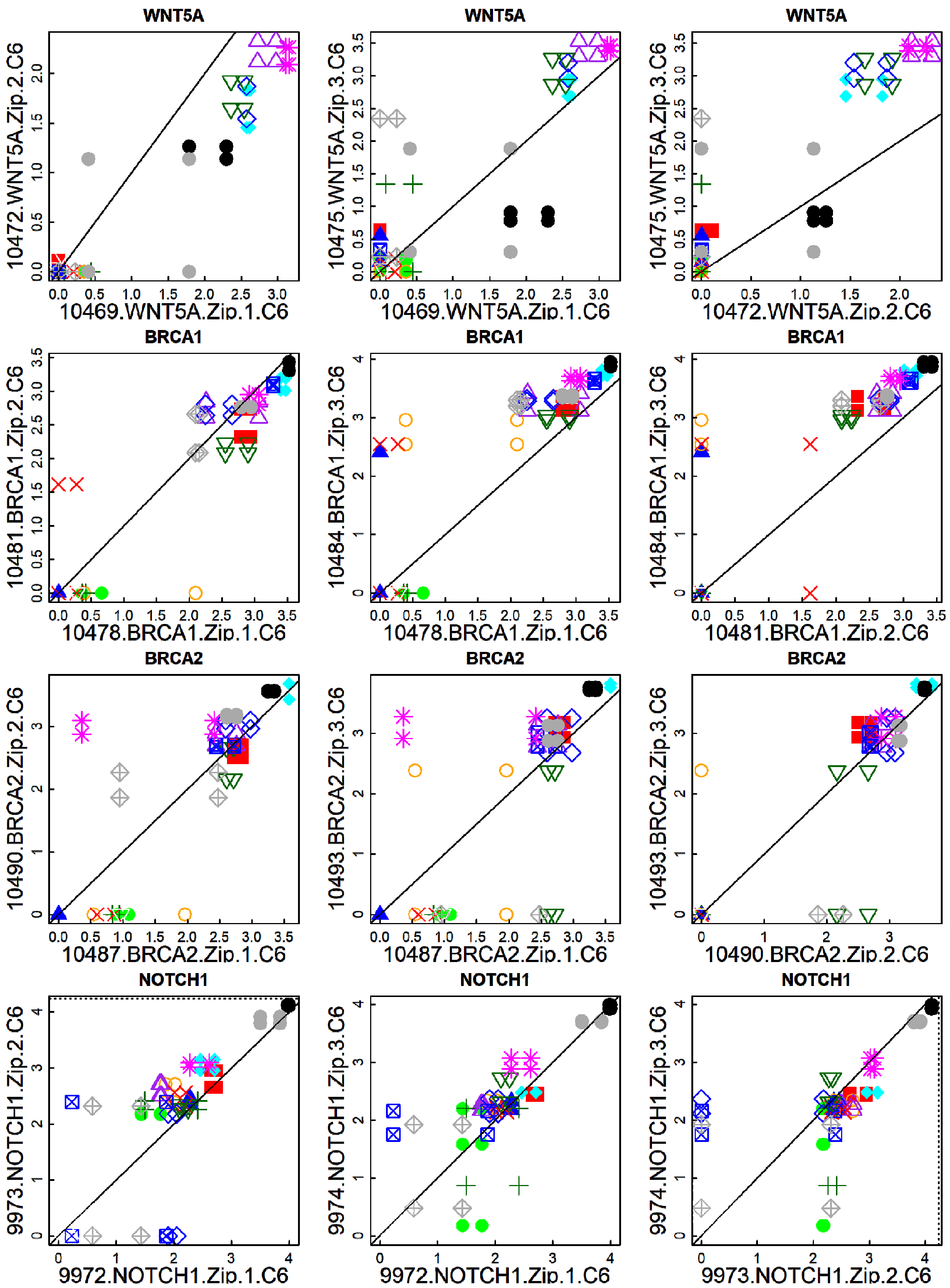
WNT5A
10472.WNT5A.Zip.2.C6
10469.WNT5A.Zip.1.C6
WNT5A
10475.WNT5A.Zip.3.C6
10469.WNT5A.Zip.1.C6
WNT5A
10475.WNT5A.Zip.3.C6
10472.WNT5A.Zip.2.C6
BRCA1
10481.BRCA1.Zip.2.C6
10478.BRCA1.Zip.1.C6
BRCA1
10484.BRCA1.Zip.3.C6
10478.BRCA1.Zip.1.C6
BRCA1
10484.BRCA1.Zip.3.C6
10481.BRCA1.Zip.2.C6
BRCA2
10490.BRCA2.Zip.2.C6
10487.BRCA2.Zip.1.C6
BRCA2
10493.BRCA2.Zip.3.C6
10487.BRCA2.Zip.1.C6
BRCA2
10493.BRCA2.Zip.3.C6
10490.BRCA2.Zip.2.C6
NOTCH1
9973.NOTCH1.Zip.2.C6
9972.NOTCH1.Zip.1.C6
NOTCH1
9974.NOTCH1.Zip.3.C6
9972.NOTCH1.Zip.1.C6
NOTCH1
9974.NOTCH1.Zip.3.C6
9973.NOTCH1.Zip.2.C6

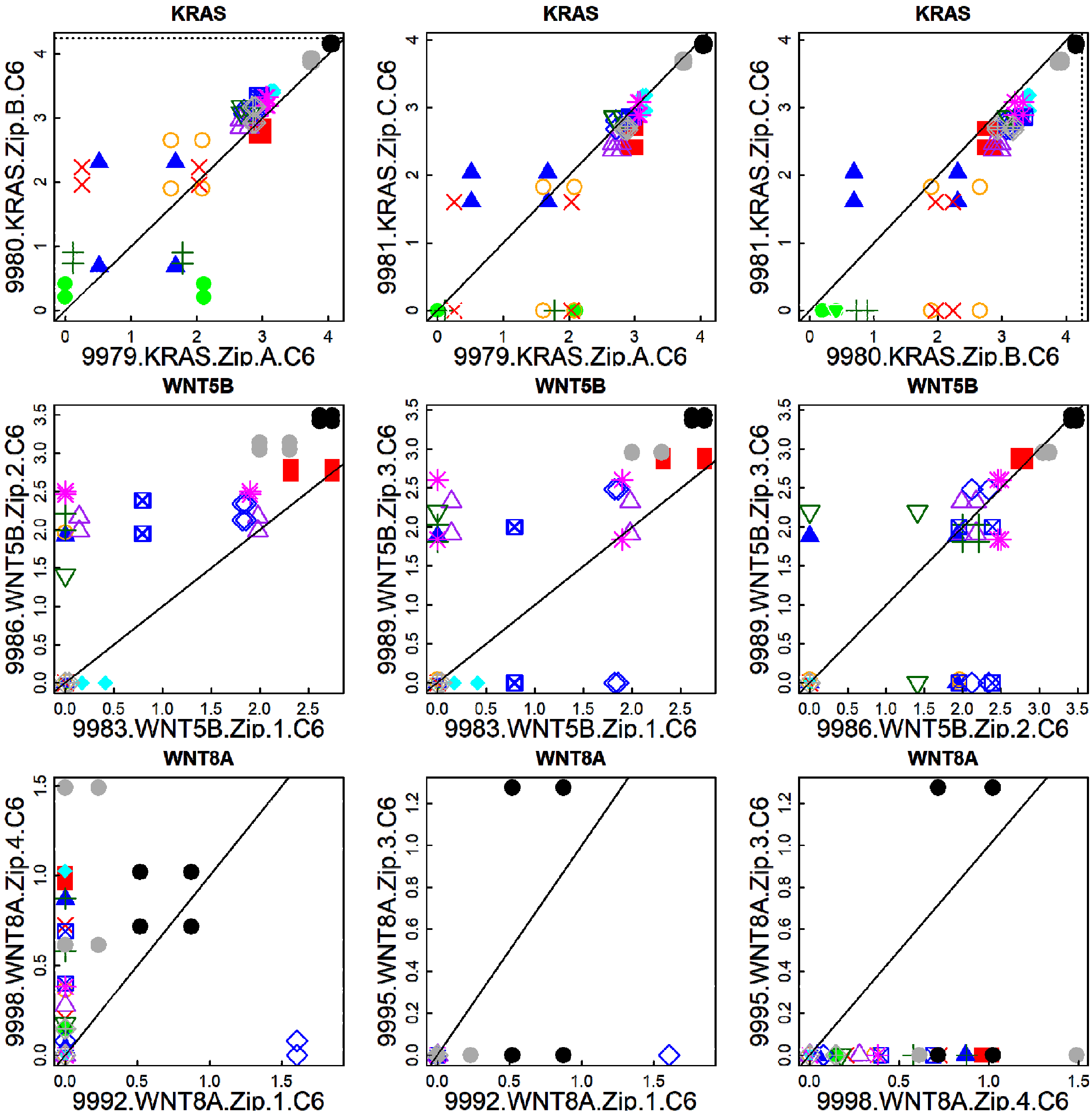
KRAS
9980.KRAS.Zip.B.C6
9979.KRAS.Zip.A.C6
KRAS
9981.KRAS.Zip.C.C6
9979.KRAS.Zip.A.C6
KRAS
9981.KRAS.Zip.C.C6
9980.KRAS.Zip.B.C6
WNT5B
9986.WNT5B.Zip.2.C6
9983.WNT5B.Zip.1.C6
WNT5B
9989.WNT5B.Zip.3.C6
9983.WNT5B.Zip.1.C6
WNT5B
9989.WNT5B.Zip.3.C6
9986.WNT5B.Zip.2.C6
WNT8A
9998.WNT8A.Zip.4.C6
9992.WNT8A.Zip.1.C6
WNT8A
9995.WNT8A.Zip.3.C6
9992.WNT8A.Zip.1.C6
WNT8A
9995.WNT8A.Zip.3.C6
9998.WNT8A.Zip.4.C6
